# Resolution of Low-Energy States in Spin-Exchange Transition-Metal Clusters: Case Study of Singlet States in [Fe(III)_4_S_4_] Cubanes

**DOI:** 10.1021/acs.jpca.1c00397

**Published:** 2021-05-28

**Authors:** Giovanni Li Manni, Werner Dobrautz, Nikolay A. Bogdanov, Kai Guther, Ali Alavi

**Affiliations:** † Department of Electronic Structure Theory, Max Planck Institute for Solid State Research, Heisenbergstraße 1, 70569 Stuttgart, Germany; ‡ Department of Chemistry, University of Cambridge, Lensfield Road, Cambridge CB2 1EW, U.K.

## Abstract

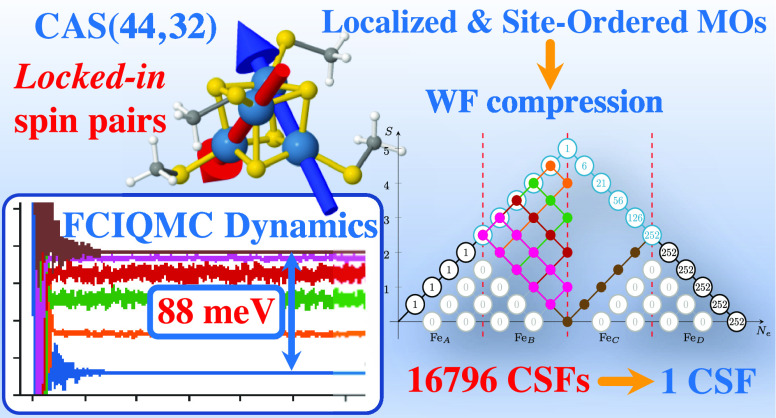

Polynuclear transition-metal (PNTM) clusters owe their catalytic activity to numerous energetically low-lying spin states and stable oxidation states. The characterization of their electronic structure represents one of the greatest challenges of modern chemistry. We propose a theoretical framework that enables the resolution of targeted electronic states with ease and apply it to two [Fe(III)_4_S_4_] cubanes. Through direct access to their many-body wave functions, we identify important correlation mechanisms and their interplay with the geometrical distortions observed in these clusters, which are core properties in understanding their catalytic activity. The simulated magnetic coupling constants predicted by our strategy allow us to make qualitative connections between spin interactions and geometrical distortions, demonstrating its predictive power. Moreover, despite its simplicity, the strategy provides magnetic coupling constants in good agreement with the available experimental ones. The complexes are intrinsically frustrated anti-ferromagnets, and the obtained spin structures together with the geometrical distortions represent two possible ways to release spin frustration (spin-driven Jahn–Teller distortion). Our paradigm provides a simple, yet rigorous, route to uncover the electronic structure of PNTM clusters and may be applied to a wide variety of such clusters.

## 1 Introduction

Polynuclear transition-metal (PNTM) clusters, such as iron–sulfur clusters and the manganese–oxygen cluster of photosystem II, play pivotal roles in biochemical processes, being crucially involved in electron transfer chains, as well as mediating spin-forbidden reactions such as oxygen evolution from splitting water.^1−12^ The catalytic activity of these compounds is to a large extent bound to the large manifold of energetically low-lying states that characterize their electronic structures. These energetically accessible electronic states allow electronic transitions—possibly varying in spin—with ease. Three different oxidation states are known for the biologically active Fe_4_S_4_ clusters, as exemplified by ferredoxins ([Fe_4_S_4_]^2+^/[Fe_4_S_4_]^+^) and the high-potential ([Fe_4_S_4_]^3+^/[Fe_4_S_4_]^2+^) proteins.^6^ An all-ferrous [Fe_4_S_4_]^0^ has also been reported.^13−15^ Only very recently an all-ferric [Fe_4_^III^S_4_]^4+^ cluster with terminal thiolates has been experimentally synthesized and characterized.^16^ Based on the experimental data, the electronic ground state of the cluster has been assigned to be a singlet. Interestingly, this oxidation state has been proposed as an intermediate state in the synthesis of the larger [Fe_8_S_7_] P-cluster core,^16^ and it has been suggested to take place in hydrophobic and sterically hindered high-potential iron–sulfur protein pockets, where it is protected against attack by nucleophiles.

At the experimental level, the ground- and excited-state electronic structures of PNTM clusters are difficult to resolve. Metal-based electronic transitions can be masked by intense ligand-based transitions, and they are part of the more complex system of vibronic excitations.^5^ Moreover, intercluster exchange interactions may exist in crystalline nonaqueous samples, and/or low-temperature measurements, affecting the magnetization measurements.^17^ Meanwhile, the theoretical characterization of these states by modern quantum chemical methods has been hindered by the computational complexity associated to the description of their ground- and excited-state wave functions. Mean-field approaches—with broken-symmetry density functional theory (BS-DFT)^18−38^ being most commonly used for these systems—fail to capture the fundamental electron correlation mechanisms involved. For example, BS-DFT is incapable to correctly describe the nonlocal correlations and fluctuations between the localized spins at the magnetic centers.^39^ However, precisely, these spin correlations are at the core of the chemical and physical properties of these compounds. Methodologies and schemes to partially circumvent the limitations in BS-DFT have been discussed extensively by Yamaguchi and co-workers,^40,41^ with *noncollinear* schemes being closer to the accurate description of the nonlocal nature of spin correlations.^42−46^ The *extended* BS-DFT represents another strategy to alleviate symmetry breaking.^38^ Exact *ab initio* wave functions, instead, allow for such correlations, but their applicability is hindered by the exponential growth of the corresponding configuration interaction (CI) expansion with respect to the number of unpaired electrons. Within *ab initio* wave function-based methods, low- and intermediate-spin states, consisting of a large number of open-shell orbitals, exhibit a considerably strong multireference character, meaning that in the CI expansion of the wave function, there are multiple electronic configurations with large relative amplitudes. Among accurate *ab initio* methods, the spin-adapted density matrix renormalization group (DMRG) approach has been widely utilized for studying exchange-coupled transition-metal clusters^11,47−51^ and applied to [Fe_4_S_4_] complexes^52,53^ with similar active spaces considered in this paper, yielding accurate energies.

The interpretation of many-body wave functions and their energetics, once available, represents another important challenge for the application of modern *ab initio* quantum chemical methods to PNTM clusters. In this respect, model Hamiltonians have been devised and used to describe the electronic structure of these systems.^54,55^

We propose a paradigm of chemically and physically motivated unitary transformations of molecular orbitals (MOs) that enables the selective targeting of energetically low-lying spin states and the effortless optimization of their CI expansions within a spin-adapted description of their wave functions. We show via theoretical and numerical arguments that the proposed unitary transformations can greatly reduce the multireference character of the wave function of these systems, thus, significantly reducing the associated computational costs. At the same time, we show that these unitary transformations allow an easy resolution of the manifold of low-lying excited states, even within the same spin symmetry sector, due to the resulting *quasi*-block-diagonal structure of the Hamiltonian matrix, thus allowing the selective targeting of one or a few of these states. Our methodology yields high accuracy comparable to DMRG studies but, crucially, allows us to obtain extremely compact forms of the many-electron wave functions, which enable immediate physical interpretation, something that is often difficult to do with other high-level *ab initio* methods.

Our paradigm represents a crucial milestone in the theoretical investigation of PNTM clusters within the first-principle quantum chemical framework. The multireference nature of the electronic wave functions of these systems represents one of the greatest challenges in modern theoretical quantum chemistry, to date believed to only be solvable in a future era of quantum computing.^56^ We demonstrate that our paradigm challenges this assumption, offering a viable theoretical route to solve the problem on classical computers with modest computational resources. Our discovery is highly advantageous for methods that exploit the sparsity of the CI Hamiltonian matrix and its eigensolutions, such as the spin-adapted full configuration interaction quantum Monte Carlo (FCIQMC)^57−67^ within the graphical unitary group approach (GUGA),^68−73^ used in this work. This paradigm provides a direct understanding of the fundamental mechanisms that govern the electron interactions and are responsible for the electronic structure of PNTM clusters.

Within the DMRG framework, localization and reordering schemes for the active MOs have long been utilized to optimally represent the local nature of electron correlation. Widely used is, for example, the Fiedler vector of the mutual information matrix.^74,75^ In this respect, our approach represents a complementary reordering scheme, motivated by the leading forms of interaction among the magnetic centers, that could also be applied to DMRG. However, no data are available in the literature indicating that localization and reordering schemes can be utilized within DMRG to *selectively* target excited states. Thus, whether DMRG can also take advantage of the block-diagonal structure of the Hamiltonian matrix and access excited-state wave functions, as shown in the present work, is still to be investigated. Considering that our scheme acts at the most fundamental level of the many-body wave function, its applicability is not limited to FCIQMC. Instead, it could also be transferred to other methodologies that operate in truncated Hilbert spaces, such as the generalized active space (GAS)^76^ and selected-CI procedures,^77−91^ as long as they are implemented within the GUGA framework.

The theoretical arguments are supported by computations on the six lowest singlet spin states of two [Fe(III)_4_S_4_(SCH_3_)_4_] cubanes in their highest oxidized form, Fe^(III)^, and with thiolate terminal ligands, an exotic form that has been synthesized only very recently.^16^ The fact that Fe(III)-based ferredoxins are dominated by local *S* = 5/2 spins has been known for decades in the inorganic chemistry community. In this work, we unambiguously show another hidden internal magnetic order for the low-energy singlet states of these compounds, namely, well-defined (locked-in) spin structures are formed within pairs of magnetic sites for all low-energy singlet states.

We also show, for the first time via *ab initio* computations, that these compounds can be mapped to the Heisenberg–Dirac–van Vleck Hamiltonian^92−94^ with two anti-ferromagnetic coupling constants. The *ab initio* results are surprisingly close to the experimental data available for one of the two structures.^16^ Although very promising, due to the simplicity of our strategy, this result must be considered cautiously. In fact, important correlation effects, such as orbital relaxation (via SCF procedure) and dynamic correlation outside the active space, are missing. Thus, it cannot be excluded that the current numerical results for the FeS cubane systems chosen are experiencing some cancellation of errors. However, the corrections arising from the missing correlation effects are quantitative in character and do not compromise the qualitative conclusions drawn in this study, which are otherwise remarkable. The presented *ab initio* results clearly indicate a specific energy ordering of the singlet spin states, strictly related to the geometrical distortions. A clear locked-in pair-magnetic ordering is observed for the two compounds. Also, the geometrical distortions are ways to release spin frustration, a phenomenon known as a spin-driven Jahn–Teller distortion.^95^

## 2 Results: Theoretical Arguments

### 2.1 Spin-Exchange-Coupled Systems

The low- and intermediate-spin wave functions of PNTM clusters, with multiple unpaired electrons at each site, are characterized by a very large number of similarly important electronic configurations. For MOs localized at the transition-metal sites, three leading classes of configurations can promptly be identified that concern the metal centers, namely, effective spin-spin interactions mediated by spin-exchange, metal-to-metal charge transfer, and excitations that violate on-site Hund’s rules (non-Hund configurations). Other important electronic configurations that contribute to the complicated electron correlation mechanisms, involve excitations from and to the bridging ligand atoms, such as ligand-to-metal charge-transfer (LMCT) excitations. A detailed analysis on the role of these terms within CI wave functions can be found in the literature.^73,96,97^

In this work, we adopt the GUGA formalism,^68−73^ which uses spin-adapted basis functions known as configuration state functions (CSFs), denoted here as |μ⟩. The total number of CSFs, *f*(*N*,*n*,*S*), dependent on the number of active electrons (*N*), orbitals (*n*), and total spin (*S*) of the wave function, combinatorially increases and is given by the Weyl–Paldus dimension formula^68^
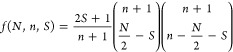
1

The FCI wave function Ψ for a given (*N*,*n*,*S*) set is written as
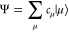
2

The sum entails the entire Hilbert space, consisting of all possible configurations, with amplitudes *c*_μ_ to be determined by the solution of the Schrödinger equation *Ĥ*Ψ = *E*Ψ via, for example, FCIQMC. Here, *Ĥ* is the quantum chemical Hamiltonian expressed in the spin-free form
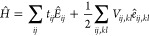
3where *t*_*ij*_ = ⟨*i*|*ĥ*|*j*⟩, *V*_*ij*,*kl*_ = ⟨*ik*|*r*_12_^–1^|*jl*⟩ are the one- and two-electron integrals of the Schrödinger operator in the chosen basis of spatial orbitals and
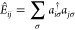
4
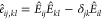
5are the spin-free excitation operators. The advantage of using this formulation in FCIQMC has been discussed in refs (72) and (73), namely, full-spin symmetry is dynamically preserved in the QMC simulation, allowing to target specific spin states.

Out of this generally vast number of configurations *f*, a smaller subset, discussed below, forms a *reference space*, which provides the leading coefficients in this expansion. If the reference space consists of only one configuration, the problem is said to be “single reference”, and the corresponding FCI wave function is generally fairly simple to approximate, for example, with standard perturbation theory. Multireference systems, with reference spaces often significantly exceeding one CSF, are much harder to handle and generally require complete-active-space (CAS)^98,99^-type methods, including FCIQMC and DMRG. Low-spin wave functions of nonmixed valence PNTM clusters, with many open-shell orbitals, fall into this category. In these systems, *spin-exchange* is the most important form of electron interactions, given by terms of the form *V*_*ij*,*ji*_. The reference space then consists of all possible distributions of spins among the singly occupied orbitals, consistent with the total spin, *S*, effectively defining a system of interacting spins, or in short, a *spin*-*system*. If there are *n*_o_ open-shell orbitals, the size of this space is given by the van Vleck–Sherman formula^100^
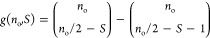
6

Although much smaller than the FCI space, *f*, the *g* space nevertheless grows rapidly with *n*_o_, making PNTM clusters extreme examples of multireference problems (more details in Section S1). We will show how the size of this reference space can be drastically reduced for wave functions of interest (such as singlet ground and low-energy excited states) through a proper selection of the used MO basis.

### 2.2 Genealogical Branching Diagrams

**Figure 1:**
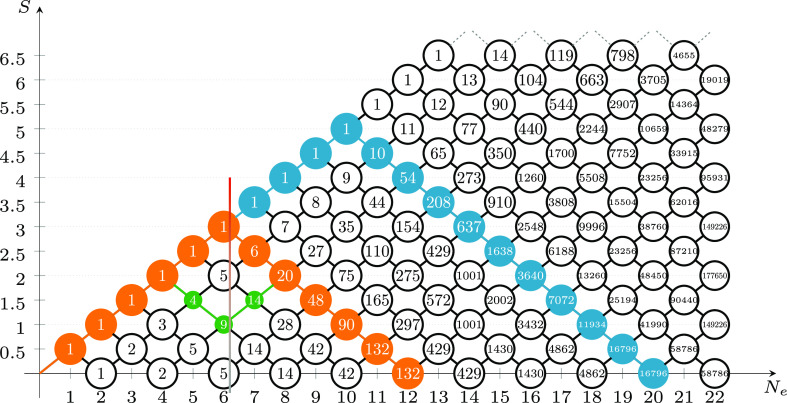
Genealogical branching diagrams describe the spin coupling of a given unpaired electron with all the previous ones in a cumulative manner. The node weights are given by the van Vleck–Sherman formula (eq 6) but can also be computed as the sum of the node weights connected from the left, indicating the number of possible paths. The orange and green paths identify two configurations out of the *g*(12,0) = 132 possible for a spin-exchange system containing 12 unpaired electrons coupled to a singlet. The green configuration is derived from the orange one by a double *spin flip*, involving orbital 5 through 8. The blue path is one of the *g*(20,0) = 16796 configurations to couple 20 unpaired electrons to form a singlet spin state.

**Figure 2:**
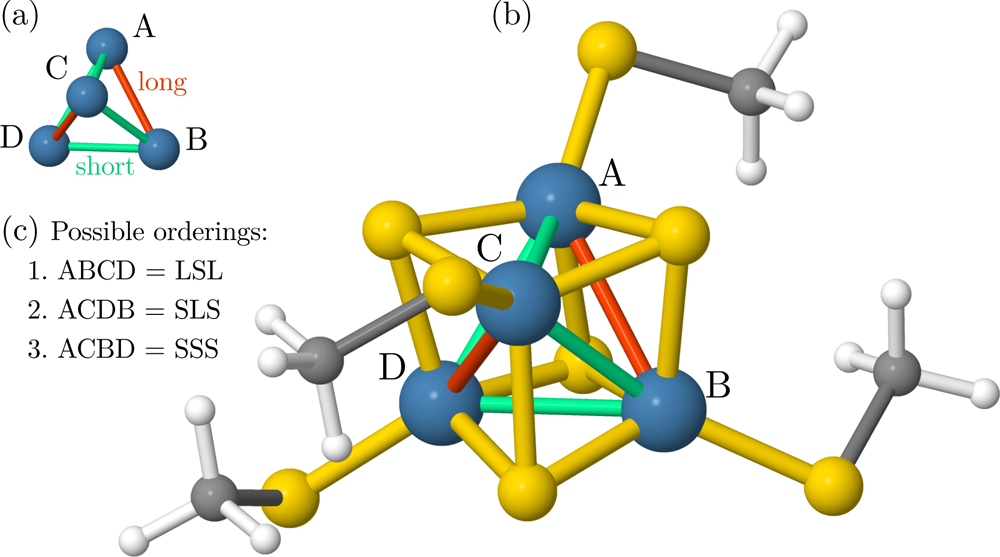
Schematic representation (a) and actual structure (b) of compound (1) used in our investigations. The magnetic centers form a distorted tetrahedron with two longer Fe–Fe bond distances (AB and CD, orange lines in (a,b), 2.846 Å) and four shorter Fe–Fe bond distances (AC, AD, BC, and BD, green lines in (a,b), 2.752 Å). In (b), white, gray, yellow, and blue spheres represent H, C, S, and Fe atoms, respectively. (c) Three possible orderings of the localized orbitals of the four magnetic centers. *L* and *S* refer to long and short bonds, respectively. ABCD is the one utilized in this work. For a perfect tetrahedron, the three orderings would be equivalent. For compound (**2**), a similar structure is considered except that system (**2**) features two short bonds (2.741 Å) and four long bonds (2.788 Å).^16^

The spin-exchange electronic configurations of spin-systems can be graphically represented via *genealogical branching diagrams*(101−106) (see Figure 1). All paths below and including the blue path in Figure 1 constitute the genealogical branching diagram of a spin-exchange model of a cluster, for example, the [Fe(III)_4_S_4_(SCH_3_)_4_] system of Figure 2, with its 20 unpaired electrons explicitly correlated in the 20 valence 3d orbitals (a (20e,20o) active space) and coupled to a singlet spin state.

The reference space of this wave function consists of *g*(20,0) = 16796 CSFs. This is the number of CSFs obtained from eq 6. A much larger space, containing *f*(20,20,0) ≈ 6 × 10^9^ CSFs, is obtained when the full CI (FCI) expansion is built from the CAS(20e,20o), which also includes configurations with doubly occupied orbitals (obtained from eq 1). Configurations with doubly occupied orbitals are not represented by genealogical branching diagrams.

While only spin-exchange interactions are assumed in the spin system, no assumptions on the actual nature of electron interactions are made in the FCI expansion, used in this work. Instead, as explained in the following, we apply MO transformations that quasi-block diagonalize the FCI Hamiltonian matrix within each spin sector. As a consequence, the spin-system character of the studied compounds directly emerges without any approximation, with the additional feature that the size of the effective reference space for a given wave function is drastically reduced.

**Figure 3:**
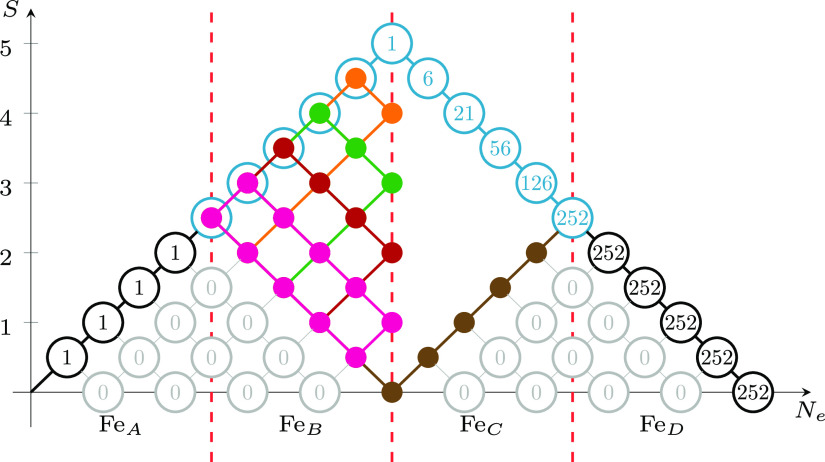
Genealogical branching diagram of a system containing four magnetic centers (Fe_A_, Fe_B_, Fe_C_, and Fe_D_), total spin *S*_total_ = 0 (singlet), and five unpaired electrons on each site with parallel spins (*S*_local_ = 5/2). The dashed red vertical lines separate four domains, each describing the spin coupling of the electrons residing on one of the four magnetic centers with all the previous ones. Gray nodes and arcs refer to non-Hund configurations which play a marginal role in the electronic wave function when the atom-separated ordering is utilized. The vanishing node weights for the gray nodes lead to the important reduction of the total number of spin-flip configurations, from a total of *g*(20,0) = 16796 (see Figure 1) to only 252 CSFs. The 252 CSFs can further be classified as: 1 for (Γ^(5)^ ⊗ Γ^(5)^) and (Γ^(0)^ ⊗ Γ^(0)^), 25 for (Γ^(4)^ ⊗ Γ^(4)^) and (Γ^(1)^ ⊗ Γ^(1)^), and 100 for (Γ^(3)^ ⊗ Γ^(3)^) and (Γ^(2)^ ⊗ Γ^(2)^), as discussed in section 2.3.

We observe a dramatic compression of the wave function—meaning far fewer CSFs populate the FCI wave functions of the targeted states—when using localized singly occupied orbitals that are sorted by magnetic centers (ABCD in Figure 2), that is, first, the five orbitals of site Fe_A_, followed by the five orbitals of each of the other sites, Fe_B_, Fe_C_, and Fe_D_. We refer to this as atom-separated ordering.^73^ In this ordering, CSFs corresponding to metal-to-metal charge transfer and non-Hund configurations will either vanish because of symmetry or contribute only marginally to the multiconfigurational expansion. For systems characterized by noncovalent bonding among magnetic sites, these configurations contribute only marginally to the wave functions of the low-energy states of PNTM clusters. However, it is only via our paradigm that their negligible contribution is fully reflected into the multiconfigurational wave functions of the low-energy spectrum of these clusters. These configurations are the ones that on site Fe_A_ do not comply with local spin *S*_local_ = 5/2. Similarly, configurations vanish that do not comply with the cumulative spin recoupling for site Fe_D_. These vanishingly small terms have been marked in gray in Figure 3. Thus, when the assumptions made upon transforming the MOs are valid, FCI eigensolvers that can take advantage of the sparsity will find these transformations very beneficial. In practice, the atom-separated ordering allows us to easily transfer known physical concepts directly to the wave function description and consequently reduces the number of leading configurations compared to the one given by the van Vleck–Sherman formula (eq 6). The localized and sorted magnetic orbitals define four domains in the genealogical branching diagram of Figure 3. The cumulative nature of the spin couplings in the genealogical branching diagrams implies that orbital ordering contributes to the overall structure of the spin-adapted representation of the wave function and ultimately to the physical interpretation of each configuration in the multiconfigurational expansion. The possibility to connect physical concepts to the control of the sparsity of large multiconfigurational expansions ultimately allows *ab initio* methods that exploit the sparsity of the CI Hamiltonian matrix and its eigensolutions, such as FCIQMC, to describe these complex electronic structures with ease.

### 2.3 PNTM Clusters as Spin-Exchange Systems

In their fully oxidized form, [Fe_4_^III^S_4_]^4+^ cubanes feature five unpaired valence electrons with parallel spin per iron center. Hence, considering these as spin systems implies the coupling of four local spins *S*_local_ = 5/2, where non-Hund configurations are excluded. Combining two spin angular momenta with local spin *S*_local_ = 5/2 results in the direct product of the six possible intermediate spin states from *S*_interm_ = 0 to *S*_interm_ = 5
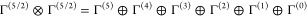
7

The two dimers further couple to give the complete wave function. In the following, we only consider the case where the two dimers are coupled to singlet spin states for the tetramer (*S*_total_ = 0). Spin couplings with *S*_total_ > 0 can be treated in a similar way.

For the *S*_total_ = 0 case, only the following direct couplings are possible
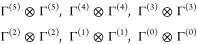


These couplings are promptly identifiable in Figure 3. The highest blue and lowest brown paths represent the (Γ^(5)^ ⊗ Γ^(5)^) and (Γ^(0)^ ⊗ Γ^(0)^) states, respectively. The (Γ^(5)^ ⊗ Γ^(5)^) case is promptly described as the anti-ferromagnetic coupling of the two dimers, (AB) and (CD), with parallel spins within each dimer. In a spin-model space of only singly occupied orbitals, the two (Γ^(5)^ ⊗ Γ^(5)^) and (Γ^(0)^ ⊗ Γ^(0)^) states are represented by a single CSF; thus, they are intrinsically single-reference (in terms of CSFs within GUGA). Of course, the expansion of the single CSF in a basis of Slater determinants leads to a multideterminantal wave function, whose coefficients are completely determined by Clebsch–Gordan coupling terms. The single-reference nature of the wave function in the CSF basis clearly shows one of the practical advantages of working in a spin-adapted basis, in addition to the possibility to target spin-pure states. On the contrary, the four intermediate states, (Γ^(4)^ ⊗ Γ^(4)^), (Γ^(3)^ ⊗ Γ^(3)^), (Γ^(2)^ ⊗ Γ^(2)^), and (Γ^(1)^ ⊗ Γ^(1)^), are intrinsically multireference within GUGA. As an example, we discuss the (Γ^(1)^ ⊗ Γ^(1)^) state in detail: there are five different paths to reach the intermediate spin, *S*_interm_ = 1 (magenta nodes, and all connecting arcs in between, in Figure 3). These five paths represent the five leading nonvanishing components in the corresponding CI expansion. Symmetrically, five paths exist for the second dimer, (CD), not drawn in Figure 3 for simplicity. Thus, the singlet spin state (Γ^(1)^ ⊗ Γ^(1)^) has a total of 25 leading CSFs. Similarly, the number of leading CSFs can be derived from Figure 3 for the (Γ^(4)^ ⊗ Γ^(4)^), (Γ^(3)^ ⊗ Γ^(3)^), and (Γ^(2)^ ⊗ Γ^(2)^) states. States (Γ^(3)^ ⊗ Γ^(3)^) and (Γ^(2)^ ⊗ Γ^(2)^) are the most multireference with 100 leading CSFs dominating their spin-exchange-only wave functions.

In a system of non-interacting magnetic centers, or with perfect cubic symmetry (*T*_d_), the six singlet states are degenerate. However, in more realistic systems, such as the [Fe_4_S_4_] cubanes studied in this work, this degeneracy is lifted because of the lowered symmetry and geometrical distortions of the molecule. In these cases, the different paths of the genealogical branching diagrams identify the leading components of the six lowest nondegenerate singlet states. The precise quantitative splittings between these states are also affected by other forms of correlations, namely, metal-to-metal charge transfer and LMCT excitations, which are obtained by diagonalizing the CAS(20e,20o) and CAS(44e,32o) problems, respectively, and will be discussed in greater detail in the next section.

**Figure 4:**
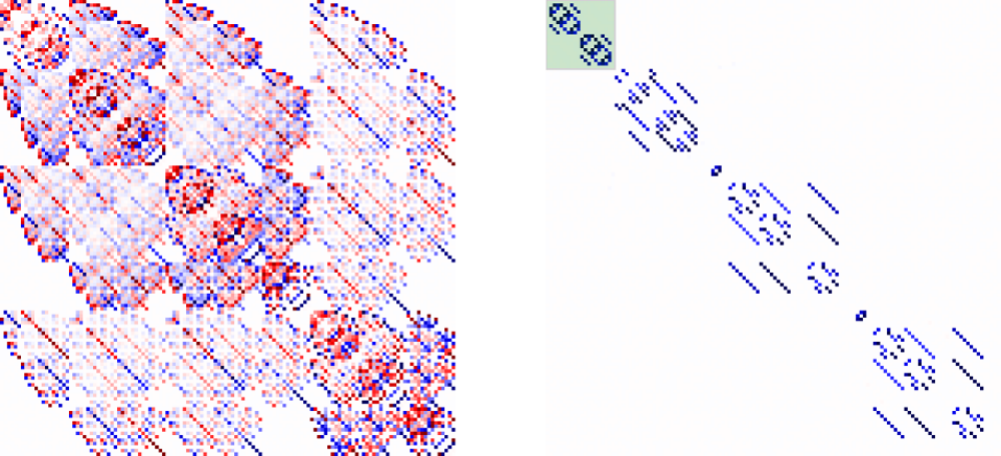
Hamiltonian matrices of exclusively exchange-coupled open-shell CSFs (including non-Hund spin-flip excitations) of a (12e,12o) active space, for an *N*_4_ model system in the same geometry as the iron atoms in Figure 2. The active space consists of the 12 2p orbitals of the nitrogens and their electrons. The Hamiltonian matrix of this simple model mimics well the one corresponding to the [Fe(III)_4_S_4_(SCH_3_)_4_] compounds, with the exception that each site features a local spin *S* = 3/2, and the intermediate pair states may only have spin *S*_AB_ ranging from 0 to 3. On the left, the orbitals are ordered as 2*p*_A_^x^, 2*p*_B_^x^, 2*p*_C_^x^, 2*p*_D_^x^, 2*p*_A_^y^,..., while on the right, the localized orbitals are ordered in the atom-separated manner described in the text. There is a striking effect on the sparsity and quasi-block-diagonal form of the CI matrix by MO localization and ordering in conjunction with a spin-adapted basis. Red and blue squares represent negative and positive Hamiltonian matrix elements, respectively. The *sign coherence* (same sign) of the Hamiltonian matrix elements that follows the atom-separated ordering is another aspect that is worth mentioning that might have important implications in understanding the *sign problem* in fermionic many-body wave functions. Nondrawn squares (white) are zero entries of the CI Hamiltonian matrix. On the right, the small 20 by 20 sub-block in the top-left corner (green background) corresponds to the CSFs depicted in Figure 3, while the remaining sub-blocks (bottom right) correspond to non-Hund spin-flip excitations.

The localization of MOs and atom-separated ordering lead to more sparse and quasi-block-diagonal CI Hamiltonian matrices. For illustration purposes, we show the block-diagonal structure of the many-body Hamiltonian in Figure 4, for the exchange-only^76^ singlet configurational space of an *N*_4_ model system (see Section S2 for details). The block-diagonal structure of the CI Hamiltonian matrix is evident. This block-diagonal structure ensures that in projective methods, such as FCIQMC, the choice of a specific CSF as the initial configuration allows us to uniquely target specific low-lying excited states within the same spin-symmetry sector. The extremely weak coupling of these initial states to the lower energy states effectively leads the projective method to converge to the lowest state matching the local spin coupling of the initial CSF, which is a particular property of the localized and ordered basis.

Thus, our proposed paradigm of MO localization and atom-separated ordering has a twofold effect on the wave functions of spin-exchange-coupled systems: (a) it provides a simple tool to compress the CI expansion of ground- and excited-state wave functions, greatly decreasing the number of leading configurations and thus computational costs. (b) It opens the route for inexpensive state-specific wave function optimizations, thus giving us the possibility to study the electronic structure of the manifold of low-lying excited states of PNTM clusters.

## 3 Results: Numerical Arguments

In this section, numerical evidence will be given of the compression and resolution of states, by considering the six low-energy singlet spin states of two [Fe(III)_4_S_4_(SCH_3_)_4_] model systems. One cubane is characterized by two long and four short Fe–Fe bonds, (**1**), the other is characterized by two short and four long Fe–Fe bonds, (**2**), as experimentally reported by Ibers and co-workers^107^ and Tatsumi and co-workers,^16^ respectively (details in Section S3). As will be evident from the discussion below, the choice of these two systems stems from their complementary geometrical distortions, which can be described as elongation and compression, for (**1**) and (**2**), respectively, along one of the *S*_4_ axis of the *D*_2*d*_ point group, which these structures belong to. From this deformation, two (or four) long and four (or two) short Fe–Fe bonds are obtained for (**1**) [or (**2**)]. Two CASs will be considered, the CAS(20e,20o) containing only the dominantly singly occupied 3d orbitals of the four iron centers and the larger CAS(44e,32o) where the additional 12 doubly occupied 3p orbitals of the bridging S atoms are also correlated (details in Section S4). The latter active space choice shows that the wave function compression and resolution of states are retained even when the FCI wave functions include forms of electron correlation (LMCT, superexchange) that go beyond the spin-exchange interactions already captured by the smaller CAS(20e,20o). Moreover, the larger active space demonstrates the trend of the spin-gap values as a function of various forms of electron correlation, which are captured by the larger active space, and not present in the smaller active space. In particular, LMCT excitations have a differential stabilizing effect on the low-spin states, thus enlarging the spin gaps among the singlet states and, as we will see in the next sections, the extracted magnetic coupling constants.

### 3.1 Resolution of the Singlet States of Compound (**1**)

#### 3.1.1 CAS(20e,20o) Wave Functions

**Figure 5:**
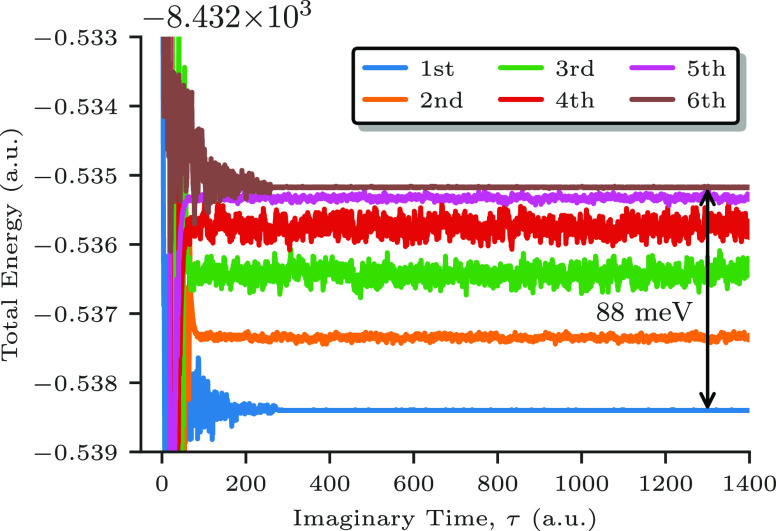
Spin-adapted FCIQMC dynamics for the six lowest singlet spin states of compound (**1**) within the CAS(20e,20o) and a walker population of 1 × 10^6^ walkers. The colors used for the trajectories correspond to the ones utilized in Figure 3 to identify the leading components of the six singlet states.

The CAS(20e,20o) spin-adapted FCIQMC trajectories for the six low-energy singlet spin states of (**1**) are reported in Figure 5, and the corresponding energy splittings are summarized in Table 1. FCIQMC solves the imaginary-time Schrödinger equation by stochastically sampling the ground- or excited-state wave function by a set of the so-called walkers. The number of walkers, *N*_w_, is a critical input parameter that determines the accuracy and the computational costs of a calculation.

**Table 1:** Energies [meV] of the Lowest Six Singlet States and the *S* = 10 State for (**1**) and (**2**), Relative to the Corresponding Ground States, as Obtained from the *Ab Initio* Calculations, the Model Hamiltonian (eq 8) Using the Extracted Parameters from Table 3, and the Experimental Data Using the Coupling Constants of Ref (16) and eq 10^a^

	Compound (**1**)				Compound (**2**)				
State	CAS(20e,20o)	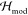	CAS(44e,32o)	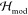	CAS(20e,20o)	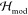	CAS(44,32o)	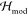	Exp.^16^
|(5, 5), 0, 0⟩	0.0	0.0	0.0	0.0	65.0	65.0	54.3	54.3	44.6
|(4, 4), 0, 0⟩	27.8	29.2		50.2	41.6	43.4		36.2	29.8
|(3, 3), 0, 0⟩	51.8	52.6		90.3	24.7	26.0		21.7	17.9
|(2, 2), 0, 0⟩	70.1	70.1		120.5	12.2	13.0		10.9	8.9
|(1, 1), 0, 0⟩	82.9	81.8		140.5	4.7	4.3		3.6	3.0
|(0, 0), 0, 0⟩	87.6	87.6	150.6	150.6	0.0	0.0	0.0	0.0	0.0
|(5, 5), 10, 0⟩	378.5	378.5	615.8	615.8	347.7	347.7	550.2	550.2	522.0

a The states are labeled |(*S*_AB_,*S*_CD_),*S*_tot_,*S*_tot_^z^⟩, as explained in the main text. It is important to note that due do the quasi-block-diagonal structure of the Hamiltonian, it is possible to target the lowest and the highest of the six singlet states directly, as opposed to conventional procedures where all states in between must be optimized, with associated considerable computational costs. This feature has been used here for the CAS(44,32) calculations.

The trajectories are rapidly converging in imaginary time and extremely stable, with stochastic fluctuations well below the energy separation among the states. Both aspects point to the fact that the wave functions are very compact with this choice of orbitals in the spin-adapted representation. Only 1 × 10^6^ walkers have been utilized for these dynamics, a tiny number in comparison with the (20e,20o) Hilbert space, and yet achieve high accuracy. Increasing the population to 1 × 10^7^ walkers has negligible effects on the lowest-to-highest spin gap (see also Section S5). For comparison, in our experience for similar systems treated with a less optimal orbital choice, a much larger number of walkers (in the range of billions) is needed to achieve a similar energy resolution.^73^

The lowest (*S*_interm_ = 5) and highest (*S*_interm_ = 0) states show the most stable dynamics, with their wave functions being dominated by the single CSFs drawn as the blue and brown paths in Figure 3, respectively, with a reference weight of 96% in both cases (see Table S5). In practice, this implies a striking operation count reduction by 5 orders of magnitude, from 16796 to 1 leading CSF. The four intermediate states, especially the ones with *S*_interm_ = 3 and *S*_interm_ = 2, show more stochastic noise, yet negligible, to be attributed to the inherently multireference nature of their wave functions within GUGA. The gap between the lowest and the highest of the six singlet states, within the (20e,20o) active space, is only 88 meV and demonstrates the level of resolution that can be obtained by MO transformations and methods that can screen out “deadwood” configurations.

The FCIQMC trajectories of Figure 5 are well-separated even though no orthogonalization procedure has been enforced, indicating the quasi-block-diagonal structure of the Hamiltonian that follows when MOs are localized and sorted by magnetic centers. The only criterion utilized to separate the states is the CSF chosen as an initial state for the FCIQMC dynamics, from which the propagation of walkers is started.

At the same level of theory, the highest spin state, *S* = 10, is 378.5 meV above the ground state (Table 1), thus suggesting that these systems are anti-ferromagnets, as already shown in ref (16), and further discussed in section 3.3 of this work.

**Figure 6:**
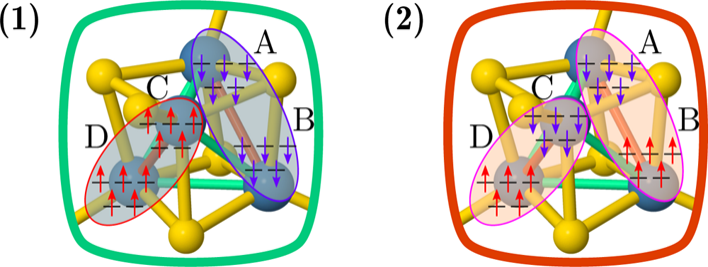
Dominant electronic configurations of the ground-state wave functions of fully oxidized [Fe(III)_4_S_4_(SCH_3_)_4_] complexes (**1**) and (**2**).

The ordering of the six singlet states and the highest *S* = 10 state can be understood by considering the geometrical distortions of the system. In (**1**), two long Fe–Fe bonds exist. In the ground-state singlet, spins along these bonds are parallelly aligned to allow an energetically favorable anti-ferromagnetic interaction within the four short bonds. On the contrary, in the highest singlet spin state, spins exhibit anti-ferromagnetic alignment along the long Fe–Fe bonds, forming two singlet AB and CD pair states (see Figures 2 and 6), thus leaving the spins along the short bonds in an energetically unfavorable uncoupled situation. We will further elaborate on this concept in section 3.3.

#### 3.1.2 CAS(44e,32o) Wave Functions

**Figure 7:**
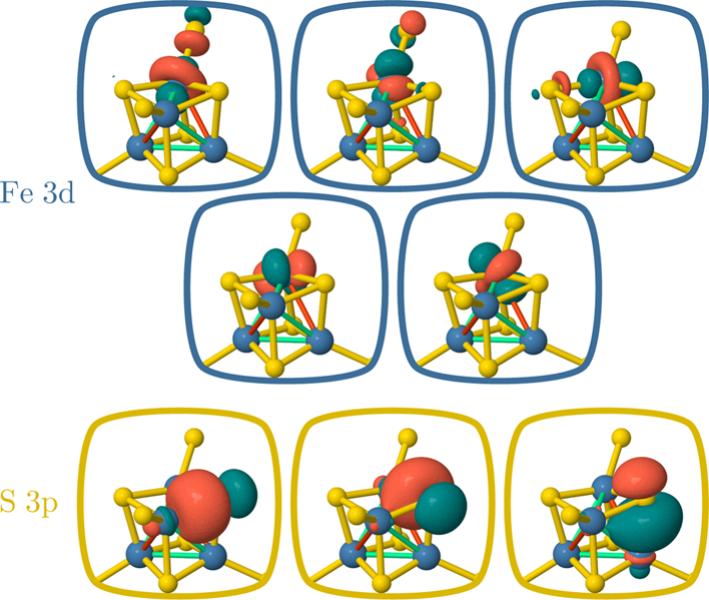
Singly occupied 3d orbitals of the Fe(III) magnetic centers of compound (**1**) (top two rows) used for the CAS(20e,20o) calculations and doubly occupied 3p orbitals of the bridging sulfur atom (bottom row) added in the enlarged CAS(44e,32o).

The doubly occupied valence 3p orbitals on each bridging sulfur atom (Figure 7) have been added to the CAS(20e,20o) active space of (**1**) to directly explore the role of ligand-mediated electron correlation effects, such as the superexchange mechanism.

The wave function compression and individual-state-resolution effects are retained also when new forms of correlation are introduced, for the main structure of the wave function is unchanged and still dominated by spin-exchange interactions. The LMCT configurations play an important role in defining the energy gap among the six singlet states. From an energetic standpoint, their role is enormous, as indicated by the nearly doubled (151 meV) energy gap between the lowest and highest singlet spin states (Table 1, see Section S5 for more details).

The role of charge-transfer excitations has also been quantified by computing the eigenvalues of the *Ŝ*_A_^2^, (*Ŝ*_A_ + *Ŝ*_B_)^2^, and (*Ŝ*_A_ + *Ŝ*_B_ + *Ŝ*_C_)^2^ spin operators (see Table 2, details in Section S6). The CAS(20e,20o) *Ŝ*_A_^2^, (*Ŝ*_A_ + *Ŝ*_B_)^2^, and (*Ŝ*_A_ + *Ŝ*_B_ + *Ŝ*_C_)^2^ eigenvalues closely follow the formal *S*_local_(*S*_local_ + 1) values to be expected from the coupling of four *S*_local_ = 5/2 spins, in line with the description of this system as a spin-system. The small deviations are to be attributed to metal-to-metal charge-transfer excitations. On the contrary, the CAS(44e,32o) wave functions show substantial deviations from the formal values both for the ground and the highest singlet states. The observed reduction of these quantities is related to effective charge-transfer excitations, from the bridging sulfur atoms to the metal centers, a form of correlation that can only be captured explicitly by the larger active space. For the CAS(44e,32o) highest singlet state (*S*_interm_ = 0), the ⟨(*Ŝ*_A_ + *Ŝ*_B_)^2^⟩ expectation value is in practice unchanged when compared to the smaller CAS(20e,20o) wave function. This is to be expected, considering that charge-transfer excitations happen symmetrically for site A and B, leading to the symmetric reduction of local spin of anti-ferromagnetically aligned centers. Upon enlarging the active space, from (20e,20o) to (44e,32o), we find a larger reduction in the ⟨*Ŝ*_A_^2^⟩ and ⟨(*Ŝ*_A_ + *Ŝ*_B_ + *Ŝ*_C_)^2^⟩ for the *S*_interm_ = 0 state as compared to the ground *S*_interm_ = 5 state. ⟨*Ŝ*_A_^2^⟩ reduces from a value of 8.69 to 7.81 for *S*_interm_ = 0, while from 8.69 to 8.34 for *S*_interm_ = 5. This aspect indicates that ligand-mediated charge-transfer effects are different for the lowest and highest singlet states and that electron interactions in these system may be more complex than predicted using a simple spin model.

**Table 2:** ⟨*Ŝ*_A_^2^⟩, ⟨(*Ŝ*_A_ + *Ŝ*_B_)^2^⟩, and ⟨(*Ŝ*_A_ + *Ŝ*_B_ + *Ŝ*_C_)^2^⟩ Expectation Values for the CAS(20e,20o) Wave Functions of the Six Singlet Spin States of (**1**)^a^

State (S_interm_)	⟨*Ŝ*_A_^2^⟩	⟨(*Ŝ*_A_ + *Ŝ*_B_)^2^⟩	⟨(*Ŝ*_A_ + *Ŝ*_B_ + *Ŝ*_C_)^2^⟩
5	8.69 (8.34)	29.77 (28.52)	8.69 (8.36)
4	8.69	19.84	8.69
3	8.67	11.88	8.67
2	8.68	5.95	8.65
1	8.69	1.99	8.69
0	8.69 (7.81)	0.02 (0.05)	8.69 (7.82)

a In parentheses, the corresponding values from the CAS(44e,32o) are reported.

The enlarged CAS(44e,32o) lowest-to-highest singlet spin gap is a clear indication of a differential role of the ligand-mediated charge-transfer excitations in the relative stabilization of the two singlet states. In the ground state, the LMCT excitations enhance the anti-ferromagnetic exchange interactions along the four short bonds. On the contrary, in the highest singlet state, the same excitations only enhance the exchange along the two long bonds. The latter enhancement is weaker, therefore leading to an overall relative stabilization of the ground state.

### 3.2 Resolution of the Singlet States of Compound (**2**)

#### 3.2.1 CAS(20e,20o) Wave Functions

The six low-energy singlet states of (**2**) show the inverted relative order compared to the same states for (**1**) (see Table 1). The ground state of (**2**) is characterized by anti-ferromagnetic alignment within each of the two short-bonded pairs, while the highest singlet state shows ferromagnetic alignment within each pair of short-bonded iron centers and anti-ferromagnetic alignment across the pairs. At the CAS(20,20) level of theory, the gap between the lowest and the highest singlet states of (**2**) is only 65 meV, while the highest spin state, *S* = 10, is 347.7 meV above the ground state.

As for compound (**1**), the relative stability of the spin states of (**2**) is promptly explained by considering the geometrical distortions of the system and anti-ferromagnetic interactions among spins of neighboring sites. In the ground state of (**2**), two short and four long Fe–Fe bonds exist, and an anti-ferromagnetic alignment of spins is observed only within the short bonds, with spins along the long bonds left uncoupled. This effect, for both (**1**) and (**2**), can be interpreted as a way to lift spin frustration and can be linked to a spin-driven Jahn–Teller distortion,^95^ which we will discuss in greater details below.

#### 3.2.2 CAS(44e,32o) Wave Functions

The results obtained for the larger CAS(44e,32o) are arresting. While the LMCT correlation effects enlarge the lowest (*S*_tot_ = 0, ground state) to highest (*S*_tot_ = 10) spin gap, as already observed for (**1**), the gap between the lowest and highest singlet states reduces, and in doing so, the energy separations among the computed states get strikingly close to the ones derived from the experimental investigation.^16^ This behavior follows from the realization that for (**2**), the LMCT excitations differentially enhance the anti-ferromagnetic exchange interactions along the four long Fe–Fe bonds that in the case of (**2**) characterize the excited singlet state. It follows that for this system, the high singlet state is differentially stabilized, and the singlet spin gap reduces.

The results discussed in this and the previous section indicate the power of our proposed strategy in providing clear qualitative trends of the electronic structures of PNTM clusters. For example, despite the simplicity of the protocol, we can promptly assess the inverted ordering of the singlet states for the two compounds that can be related to geometrical distortions. Although important correlation effects are still missing from our protocol, such as orbital relaxation and dynamic correlation effects outside the active space, we clearly show that for this class of anti-ferromagnetically coupled systems, various forms of leading correlation mechanisms favor the anti-ferromagnetic coupling, thus enlarging the gap among the singlet states for (**1**) and interestingly reducing the gap for (**2**), as larger active spaces are considered, and more electron correlation is accounted for. However, these effects are quantitative in nature and do not affect the qualitative trends discussed in this work.

### 3.3 Exchange Interactions via a Model Hamiltonian

In order to support and rationalize the *ab initio* results, we utilize the isotropic Heisenberg-Dirac-van Vleck model Hamiltonian with nearest-neighbor interactions^92−94^
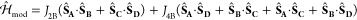
8where *J*_2B_ and *J*_4B_ are two nonequivalent coupling constants, following the geometric distortions of the Fe_4_S_4_ systems (Figure 2). **Ŝ_*i*_** (with *i* = A, B, C, and D) are local spin 5/2 operators. This model Hamiltonian has also been chosen in ref (16). For (**1**), *J*_2B_ and *J*_4B_ refer to the two long and the four short bond interactions, respectively. For (**2**), the two coupling constants refer to the two short and the four long bonds, respectively. The matrix elements of the Hamiltonian (eq 8) can be computed in the uncoupled basis defined by local spin projections, |*S*_A_^z^*S*_B_^z^*S*_C_^z^*S*_D_^z^⟩, using the ladder-operator expansion for each **S**_*i*_. The corresponding Hilbert space consists of 1296 states. A unitary transformation, *Û*, to the total-spin-adapted coupled basis can be constructed using the relevant Clebsch–Gordan coefficients ⟨*j*_1_*m*_1_*j*_2_*m*_2_|*jm*⟩. Its elements are given by
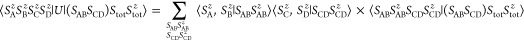
9where *S*_AB_ and *S*_CD_ refer to spin operators over the AB and CD pairs of iron centers and *S*_tot_ and *S*_tot_^z^ are the target total spin, in this case, *S*_tot_ = 0, and its projection (indicated by the *z* superscript). The choice of the spin coupling scheme is important because  takes a diagonal form in the |(*S*_AB_*S*_CD_)*S*_tot_*S*_tot_^z^⟩ basis, while it only has a block-diagonal structure if other spin coupling schemes are used, such as |(*S*_AC_*S*_BD_)*S*_tot_*S*_tot_^z^⟩ or |(*S*_AB_*S*_ABC_)*S*_tot_*S*_tot_^z^⟩. We want to emphasize that the diagonal structure in the coupled basis is a consequence of the symmetry of the system. The corresponding configurations dominate the low-energy eigenstates both of the model and the *ab initio* Hamiltonian as discussed in the previous sections. It is tempting to suggest the spin coupling of eq 9 (coupling A to B and C to D prior to the coupling of AB and CD pairs) also for the *ab initio* Hamiltonian within the FCIQMC dynamics, in order to make the four internal states (from *S*_interm_ = 1 to *S*_interm_ = 4) single-reference. However, as discussed in section 2 of this work and in our earlier work,^73^ this is not possible within the GUGA framework where a cumulative coupling scheme is utilized. Whether a more general framework exists that allows efficient Hamiltonian matrix element evaluations in noncumulative spin coupling schemes is a question that remains to be answered.

Also, as shown by our numerical results, further compression is not needed within FCIQMC, as the stability of the dynamics is already satisfactory. The gauge utilized of coupling orbitals in cumulative order is at the core of the GUGA procedure to efficiently calculate Hamiltonian matrix elements, also within the stochastic framework.

An analytical expression for the eigenvalues of the model Hamiltonian (eq 8) was obtained by Griffith^108^
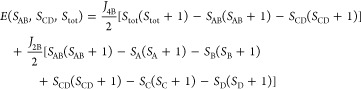
10where the *S*_*i*_ on each metal site are kept to the constant value of 5/2 for the Fe(III) case. The energies of the six possible states of the *S*_tot_ = 0 spin sector, parameterized in the exchange coupling constants, are (*J*_4B_ – *J*_2B_) × {0, 10, 18, 24, 28, 30}, for the states with intermediate spins *S*_AB_ = *S*_CD_ = {5, 4, 3, 2, 1, 0}, relative to the |(5, 5), 0, 0⟩ state. The splittings within the singlet manifold are completely defined by the difference of the exchange parameters, (*J*_4B_ – *J*_2B_), while the high-spin, *S*_tot_ = 10, |(5, 5), 10, 0⟩ state is 55*J*_4B_ above the singlet |(5, 5), 0, 0⟩ state.

We can evaluate the exchange parameters by mapping the model eigenvalues to the corresponding *ab initio* excitation energies. Using the three |(5, 5), 0, 0⟩, |(0, 0), 0, 0⟩, and |(5, 5), 10, 0⟩ states, the exchange parameters of Table  3 are obtained. The energy splittings of the six low-energy singlet states, obtained from the parameterized model, are reported in Table 1.

**Table 3:** Parameters [cm^–1^] of the Model Hamiltonian (eq 8) Extracted from the *Ab Initio* Calculations for Compounds (**1**) and (**2**), and the Experimentally Obtained for (**2**) from Ref (16)

	Compound (**1**)		Compound (**2**)		
	(20e,20o)	(44e,32o)	(20e,20o)	(44e,32o)	Exp.^16^
*J*_4B_	55.5	90.3	41.5	72.7	70
*J*_2B_	32.0	49.8	58.9	87.3	82
*J*4B – *J*2B	23.5	40.5	–17.5	–14.6	–12

For (**1**) and (**2**), both coupling parameters are anti-ferromagnetic, demonstrating the intrinsic frustration in these systems. In compound (**1**), the larger *J*_4B_ interaction forces spins along the longer bonds to be ferromagnetically aligned (Figure 6). The elongation of the Fe_A_–Fe_B_ and Fe_C_–Fe_D_ bonds is a direct consequence of the unfavorable interaction of the frustrated spins. For (**2**), (*J*_4B_ – *J*_2B_) < 0 confirms the inverted relative ordering of the six states. The agreement between the *ab initio* and the model Hamiltonian results is exceptionally good, despite the simplicity of the latter. Moreover, the model Hamiltonian provides an estimate for states that are harder to obtain within the *ab initio* framework.

The small deviations between the model and *ab initio* Hamiltonian (∼2 meV) may be attributed to the absence of higher-order couplings in the model Hamiltonian, such as the biquadratic and ring exchange.^92,109−113^ It should also be noticed that more rigorous methods to extract model parameters, based on the construction of an effective Hamiltonian, where both *ab initio* energies and wave functions are utilized, have been proposed in the literature.^114^ Nevertheless, taking higher-order couplings into account and using more rigorous extraction procedures would lead only to marginal changes in the extracted parameters, when the current *ab initio* results are utilized for the mapping. More important are the changes that might follow from improvements of the *ab initio* description. In fact, we need to be cautious about the surprisingly good agreement of the *ab initio* and experimental results. Our approach does not account for important correlation effects, such as orbital relaxation effects, generally captured by CASSCF procedures, and dynamic correlation, generally accounted for by post-CASSCF methods. Thus, it is possible that the quantitative matching follows from some cancellation of errors. Results on the effect of orbital relaxation will be presented in a separate study. In the absence of more accurate *ab initio* computations, it is hard to make any more conclusive judgment on the possible role and magnitude of higher-order forms of magnetic interactions.

The coupling constants extracted from the CAS(44e,32o) *ab initio* calculations are larger compared to the ones obtained from the CAS(20e,20o). However, as shown in Table 3, the increase is different for the two coupling constants, indicating that the LMCT excitations (explicitly included in the larger active space) have a differential enhancing effect on the superexchange mechanism for the long and short bonds. For (**1**), the anti-ferromagnetic exchange interactions among the four short bonds is enhanced by the LMCT more than the one between the two long bonds. Thus, *J*_4B_ increases more than *J*_2B_ in (**1**) and the (*J*_4B_ – *J*_2B_) value is enlarged (from 23.5 to 40.5 cm^–1^, Table 3). On the other hand, for (**2**), the anti-ferromagnetic interaction along the two short bonds is differentially enhanced, *J*_2B_ grows more than *J*_4B_, and ultimately, the (*J*_4B_ – *J*_2B_) value is reduced.

The effect on energetics and extracted magnetic coupling parameters, arising from the LMCT excitations, which are explicitly considered in CAS(44,32) and missing in CAS(20,20), is surprising: LMCT excitations differentially stabilize the low-spin states (*S* = 0) over the high-spin state (*S* = 10), and they further enlarge the spin gaps among the six singlet states for compound (**1**), while reducing the gap among the six singlet states of (**2**). As a consequence, spin-state energetics and magnetic coupling parameters of compound (**2**) become surprisingly close to the available experimental data, with *J*s only within a few wavenumbers. While these results are very promising and qualitatively arresting, it is possible from a quantitative standpoint that other forms of electron correlation, not yet considered in our protocol, will further change the spin-state separation. This effect, however, is expected to be only quantitative. The qualitative conclusions discussed in this work will remain. Namely, the relative spin-ordering and its inversion as a function of the geometrical distortion, and the fact that superexchange mechanisms for these systems favor anti-ferromagnetic interactions between magnetic centers.

The observation that the *J*_4B_ = *J*_2B_ model, which corresponds to a model with perfect tetrahedral symmetry, gives rise to a sixfold degenerate singlet states has interesting consequences. Griffith^108^ has shown that in the case of *S* = 5/2 Heisenberg model, the six singlet states span *A*_1_ + *A*_2_ + 2*E* irreducible representations. The presence of states with *E* symmetry implies the possibility of a spin-driven Jahn–Teller distortion, where the degeneracy is lifted by distortions of *E* symmetry, contained by the symmetric square (E ⊗ *E*)_+_ = *A*_1_ ⊕ *E*. Similar considerations will apply to the higher spin states as well, where degeneracies of other types (*T*_1_ and *T*_2_) occur and which may be lifted by distortions of *T*_2_ symmetry. Thus, vibronic effects on the spectra of such cubanes may be understood and predicted as the interplay between spin-frustration and Jahn–Teller distortions, a point we return to in a separate publication. Interestingly, these distortions already exist in homovalent all-ferric [Fe(III)_4_S_4_(SCH_3_)_4_] clusters and are not to be related specifically to the mixed-valence species. In this context, more involved model Hamiltonians which include vibronic coupling effects and their coupling with the electronic states have been previously studied extensively for ferredoxins by Bominaar and co-workers^115,116^ and show complex interplay between the electronic structure and geometry.

## 4 Discussion

We propose a paradigm consisting of simple and physically motivated MO transformations (localization and reordering) that, in realistic spin-exchange-coupled PNTM clusters, lead to FCI molecular Hamiltonian matrices within the GUGA formalism with an extraordinary quasi-block-diagonal structure. The spin-system nature of these clusters directly emerges from this structure of the Hamiltonian, without any simplifying approximation. A large compression of the multiconfigurational wave functions follows, which can be understood via simple genealogical branching diagrams. Moreover, the quasi-block-diagonal structure of the Hamiltonian opens the route to direct state-specific wave function optimizations of ground and excited states, thereby removing the often undesired overhead of computing all intermediate states. Methods such as FCIQMC greatly benefit from these features, enabling accurate calculation of the wave functions with modest computational effort. Considering the fundamental nature of our finding, selective optimization of excited-state wave functions is to be expected within other methodologies; examples are given by truncated CI procedures, such as the GAS approach^76^ and selected-CI schemes.^77−91^ The construction and spin coupling within GUGA (or other spin-adapted bases) is a condition to the success of the state-specific optimization within spin-exchange-coupled PNTM clusters. The GUGA spin adaptation has already been utilized for the GAS approach.^76^ Also, the suggested scheme could represent an alternative MO reordering protocol within DMRG that reduces entanglement and improves convergence with the bond dimension parameter (*M*), an alternative to the already widely used schemes within DMRG, such as the Fiedler vector approach of mutual information matrix.^74^

This paradigm allows us to unravel the complicated electronic correlations in PNTM clusters and to provide straightforward physical interpretations of the magnetic interactions within. This is demonstrated in the case of two fully oxidized [Fe(III)_4_S_4_(SCH_3_)_4_] clusters, by investigating the magnetic interactions in their six energetically low-lying singlet states. Highly compressed (*S*_interm_ = 1 to *S*_interm_ = 4) and even single-reference (*S*_interm_ = 0 and *S*_interm_ = 5) wave functions are obtained that allow a simple physical interpretation of the magnetic interactions characterizing these systems. The residual multireference character of the internal singlet spin-states (from *S*_interm_ = 1 to *S*_interm_ = 4) is to be attributed to the cumulative spin coupling utilized within the GUGA framework, and in principle, they can also be made single-reference by unitary transformation of the GUGA basis or by directly optimizing the wave functions within a different spin coupling scheme, as exemplified by eq 9 for the Heisenberg model. However, it is precisely the cumulative nature of spin couplings that makes the GUGA approach efficient, of general applicability, and thus practical. Whether a mathematical framework exists that allows efficient evaluation of Hamiltonian matrix elements for general spin couplings is a question that remains to be answered. It is our hope that this work could create momentum for directing research in various areas/methodologies of quantum chemistry, in this direction.

Our results show that a *hidden* magnetic order exists in this manifold of states, that is, well-defined spin structures are formed within two pairs of magnetic centers, which are subsequently coupled to form one of the six singlet spin states. Namely, for the ground-state of (**1**), an S_interm_ = 5 coupling within the AB and CD pairs is obtained, which then couple to form an overall singlet spin state. Similarly, for the highest singlet spin states of (**1**), the same pairs are coupled to the intermediate singlet, *S*_interm_ = 0. For compound (**2**), this intermediate magnetic ordering is inverted; for the ground state, we find *S*_interm_ = 0 for AB and CD pairs, and *S*_interm_ = 5 for the same pairs, in the case of the highest singlet spin state. The fact that Fe(III)-based ferredoxins are dominated by local *S* = 5/2 spins has been known for decades in the inorganic chemistry community, starting from the works by Anderson and Hasegawa in the 1950s,^117^ followed by the pioneers of the study of iron–sulfur systems, such as Mouesca and Lamotte^118^ and Girerd and Blondin.^119^ In fact, the origins are also deeply connected to ligand field theory and spectroscopy.^120^ However, the fact that these systems show specific (*locked-in*) spin couplings within the AB and CD pairs, already predicted by Griffith^108^ in the framework of the Heisenberg model, emerges in our work for the first time. This is not to be confused with the locked-in spin couplings among pairs in mixed-valence systems, where specific spin couplings are enforced by the delocalization of the extra electrons.

The agreement between the quantum chemical predictions deriving from our protocol and the experimental measurements is impressive, with coupling constants in agreement within a few wavenumbers. While promising, the numerically good agreement needs to be considered cautiously. In fact, while for *S* = 1/2 spin systems, the Heisenberg Hamiltonian consisting of the simple bilinear exchange interactions is acceptable, for the coupling of multiple S ≥ 1 local spins biquadratic exchange interactions are generally necessary to account for isotropic deviations from the bilinear model.^113^ Owing to the exceptionally good agreement between the *ab initio* and the eigenvalues of bilinear Heisenberg Hamiltonian in this work, the biquadratic constants also obtained from the fitting procedure were in practice negligible. Albeit more rigorous methods to extract model parameters from *ab initio* data based on the construction of an effective Hamiltonian have been proposed in the literature,^114^ deviations from the bilinear Heisenberg model are to be expected already at the level of the *ab initio* eigenvalues, as soon as important correlation effects, such as orbital relaxation and dynamic correlation effects, missing in the present protocol, would be included. In the absence of more accurate *ab initio* computations, it is hard to make any more conclusive judgment on the possible role and magnitude of higher-order forms of magnetic interactions and whether the matching is fortuitous or not.

Considering the general applicability of our paradigm, the implications are in practice far-reaching. This methodology can be applied to a wide range of PNTM clusters, including [MnO] and [CoO] cubanes, to partially reduced systems, and different spin states, and will be of great value in uncovering the chemical activity of these systems in electron transport, oxygen evolution, and potentially other spin-forbidden reactions. Moreover, the proposed paradigm is not bound to any specific methodology, for example, FCIQMC. Instead, it can be applied to any many-body wave function eigensolver that can take advantage of the resulting block diagonal structure of the Hamiltonian and the sparsity of the corresponding wave functions. Our results demonstrate via theoretical and numerical arguments that the paradigm we are proposing is a crucial milestone in the application of many-body quantum chemical procedures to the complicated electronic structures of PNTM clusters.

## Supporting Information Available

The following topics are covered in the Supporting Information: Comparison of the exponential scaling of the many-body wave function based on all combinatorially possible CSFs (Weyl–Paldus formula, eq 1), and only exchange-coupled configurations, based on the van Vleck–Sherman formula (eq 6) (Section S1); some details on how we construct Hamiltonian matrices of exclusively exchange-coupled open-shell CSFs based on the GAS framework (Section S2);^76^ geometrical details of (**1**) and (**2**) (Section S3); additional computational details (basis set, orbitals, and active space) (Section S4); details on the FCIQMC dynamics (population bias) and analysis of the optimized wave functions (Section S5); and details on local spin measurements (Section S6) (PDF)
