# Supplementary material for: Resolution of Low-Energy States in Spin-Exchange Transition-Metal Clusters: Case Study of Singlet States in [Fe(III)4S4] Cubanes
Source: J Phys Chem A. 2021 May 28;125(22):4727–40. doi: 10.1021/acs.jpca.1c00397 (PMC8201447; doi:10.1021/acs.jpca.1c00397)
Supplement: Supplementary file 1 — jp1c00397_si_001.pdf [file jp1c00397_si_001.pdf]

Supplementary Material: Resolution of Low-Energy States in Spin-Exchange Transition-Metal Clusters: Case Study of Singlet States in $[\mathrm{Fe(III)_4S_4}]$ Cubanes

Giovanni Li Manni,*,† Werner Dobrautz,† Nikolay A. Bogdanov,† Kai Guther,† and Ali Alavi†,‡

†*Department of Electronic Structure Theory, Max Planck Institute for Solid State Research, Heisenbergstraße 1, 70569 Stuttgart, Germany*

‡*Department of Chemistry, University of Cambridge, Lensfield Road, Cambridge, CB2 1EW, United Kingdom*

E-mail: G.LiManni@fkf.mpg.de

Contents

S1 Exponential scaling explained **S2**

S2 Construction of exchange-coupled Hamiltonian matrices **S4**

S3 Geometrical details **S5**

S4 Basis set, orbitals and active spaces **S9**

S5 GUGA-FCIQMC details **S10**

S6 Local spin measurements **S14**

References **S15**

S1 Exponential scaling explained

For readers not familiar with the topic we offer in Figure S1 a visualization showing the exponential scaling of the number of CSFs in a FCI expansion (green circles), according to the Weyl-Paldus formula (Equation 1 in the main document), and in a space made only of exchange-coupled configurations (a spin-system, red triangles), according to the van Vleck-Sherman formula (Equation 6), as a function of the number of correlated electrons (N) and orbitals (n). For simplicity we have set $n = n_O = N$ and coupled the electrons to a singlet spin state ($S = 0$). The computational limit of a FCI optimization, using conventional methods,[1,2] is in practice already reached when around 18 electrons are fully correlated in 18 orbitals, CAS(18e,18o). Although the number of CSFs in a spin-system grows slower than the corresponding FCI expansion, the computational limit to perform a CI optimization in such space is reached when about 36 electrons are correlated in 36 orbitals. These limits are indicated by the gray shaded area in Figure S1.

The main computational bottleneck for a conventional optimization of the CI coefficients is the necessary storage of at least three vectors of the size of the Hilbert space of the problem, needed in iterative solvers like the Davidson[1] or the Lanczos algorithm.[2] Assuming 8 byte memory requirement for a single entry in these CI vectors, one already reaches $\sim$ 635GB for a CAS(22e,22o) calculation, with $S = 0$, for each vector.[3,4]

Interestingly, the limit estimated for a spin-system with 36 electrons in 36 orbitals makes the optimization of the Fe_8S_7 core of any P-cluster unfeasible, via exact diagonalization procedures, even if the system is treated as a simple spin-system and the active space consists

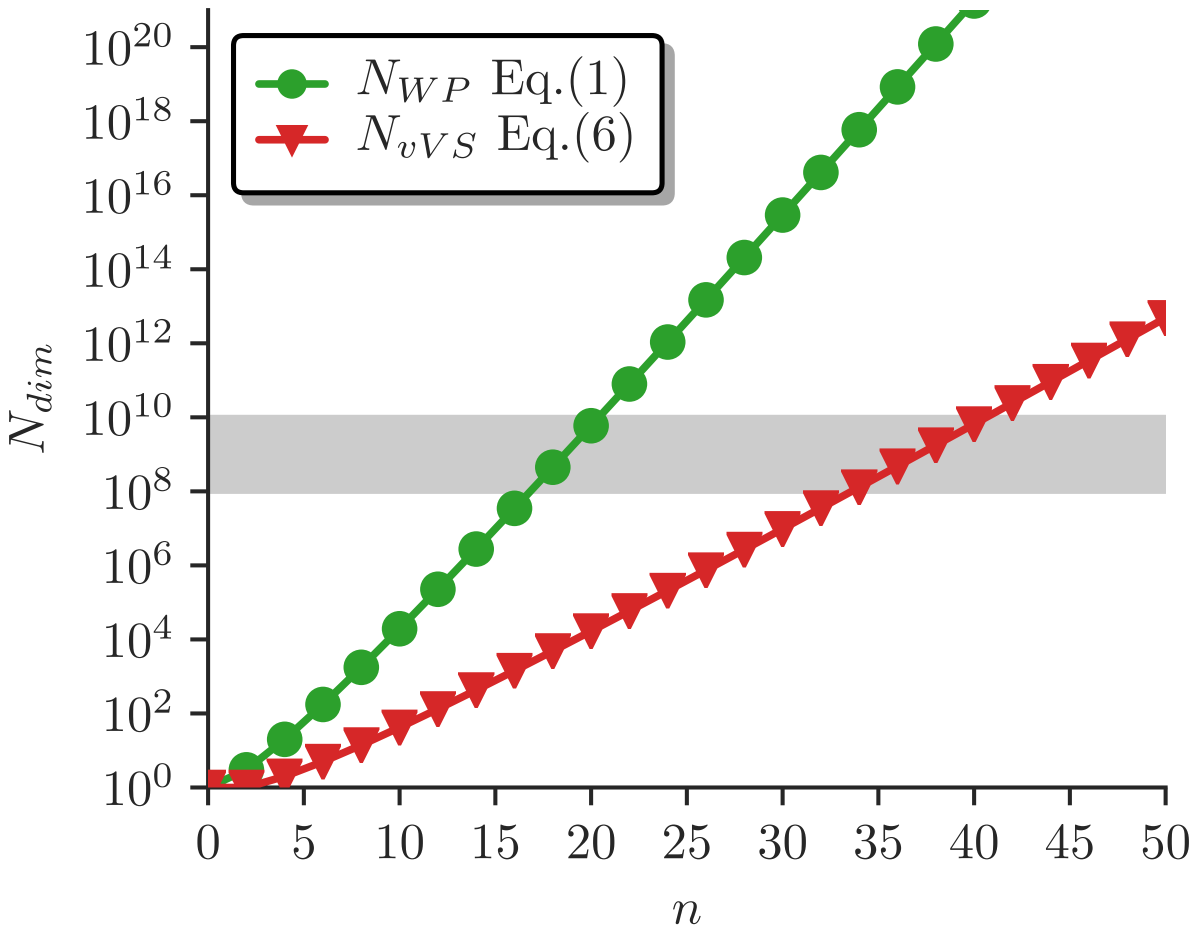

Figure S1: Number of CSFs according to the Weyl-Paldus formula (Equation 1) and the van Vleck formula (Equation 6) as a function of the number of spatial orbitals $n = n_O = N$ for $S = 0$. The gray area indicates the zone where exact diagonalization procedures require significant computational resources. Beyond the gray area computations are considered unfeasible with current hardware/software technology.

only of the 3 d electrons and orbitals on each site, a CAS(40e,40o) for the super-oxidized species. As discussed in the main document, via our paradigm an astonishing compression of the wave function is obtained, greatly reducing the number of leading CSFs, even when compared to the already relatively small space of exchange-coupled CSFs. We believe our paradigm will give us access to the optimization of the P-clusters states. This will be the main focus of our future research.

S2 Construction of exchange-coupled Hamiltonian matrices

In this section we provide more details on how we construct Hamiltonian matrices of exclusively exchange-coupled open-shell CSFs, for chosen active spaces containing only unpaired electrons. This method has been used to build the matrix depicted in Figure 4, and it is further discussed in our previous work.[5]

We use a technology, known as the *Generalized Active Space* (GAS) approach, firstly proposed by Jeppe Olsen,[6] and since 2011 made available in the Molcas and the OpenMolcas chemistry software packages, within the multiconfigurational self-consistent field (GASSCF) framework and with full spin-adaptation using GUGA.[7]

GAS wave functions are built by defining a number of active orbital subspaces; within each subspace, all possible electron excitations are allowed, while having the possibility to enforce restrictions on the number of inter-space excitations. GAS spaces are defined "disconnected" if no excitations among them are permitted, while they are said "connected" if inter-space excitations are permitted. In the same GAS wave function, both connected and disconnected spaces can exist. Although highly constrained, GAS wave functions with disconnected spaces are of great theoretical and practical interest. For example, disconnected spaces can be utilized to selectively forbid inter-space excitations that can be related to specific electron-correlation mechanisms, such as charge-transfer (CT) excitations.

As for the other systems discussed in this work, the MOs of the tetrahedral N_4 system, discussed in Figure 4, have been localized and ordered by atom prior the choice of the GAS spaces. For the construction of the GAS wave function, 12 orbital subspaces have been chosen, one for each of the twelve 2 p orbitals of the system. Only one electron is allowed per GAS subspace, thus, eliminating any CT excitation. These restrictions immediately lead to a space of purely exchange-coupled CSFs. It is important to highlight that in cases where covalent bonds are expected, CT configurations in a basis of localized orbitals will dominate the FCI wave function. Removing them from the many-body expansion will negatively affect the optimized wave function, meaning the resulting wave function will not reflect the true physical and chemical interactions (including the actual bonding) of the system. On the contrary, when CT excitations are negligible the disconnected GAS wave functions can capture the spin-system nature of the structure investigated. The case of the N_4 model system falls into this category. Another aspect that it is worth mentioning is the realization that the GAS Hamiltonian matrix of Figure 4 in *atom-separated order* shows *sign coherence* throughout all its elements, meaning all elements of the matrix on the right of Figure 4 are negative, as opposed to the matrix on the left that contains both positive and negative entries. This properties is still under investigation, however, it might have important implications in understanding the *sign problem* in fermionic many-body wave functions.

S3 Geometrical details

In Table S1 and Table S2 we report the Cartesian coordinates of (**1**) and (**2**), respectively. Structure (**1**) is the one from Reference 8, which is an optimized BS-DFT geometry using the BP86 functional and a triple zeta valence basis set.[9] The two long (2.85 Å) and four short (2.75 Å) Fe−Fe bonds are the relevant geometrical elements for our work, because the spin gaps among the singlet spin states directly bound to them. Two long and four short bond lengths were already reported by Ibers and co-workers in their original experimental

Table S1: Cartesian coordinates [Å] of (**1**).

Atom	X	Y	Z
Fe1	0.05	-1.37	1.01
Fe2	-1.38	0.05	-1.00
Fe3	-0.05	1.38	1.00
Fe4	1.37	-0.05	-1.01
S5	0.04	-1.78	-1.29
S6	-0.04	1.78	-1.29
S7	1.78	-0.04	1.29
S8	-1.78	0.04	1.29
S9	0.24	3.30	2.14
S10	-0.24	-3.29	2.14
S11	-3.29	-0.24	-2.14
S12	3.29	0.24	-2.14
C13	-3.80	-1.84	-1.38
H14	-3.91	-1.71	-0.29
H15	-4.76	-2.17	-1.81
H16	-3.03	-2.60	-1.56
C17	3.80	1.83	-1.38
H18	3.91	1.71	-0.29
H19	4.76	2.16	-1.81
H20	3.03	2.59	-1.55
C21	-1.83	-3.80	1.38
H22	-2.16	-4.76	1.81
H23	-2.59	-3.03	1.55
H24	-1.70	-3.91	0.29
C25	1.84	3.80	1.38
H26	2.17	4.76	1.81
H27	2.60	3.03	1.56
H28	1.71	3.91	0.29

work.[10] Interestingly, in their work the difference between the long (2.776 Å) and the short (2.732 Å) bond distances was sensibly smaller compared to the one reported by Sharma and coworkers.[8] Based on our findings, this crucial difference implies that for the experimental structure of Ibers and co-workers a smaller (J_{4B}-J_{2B}) value, and thus smaller energy splittings among the singlet states, are to be expected.

Structure (**2**) has been derived from Reference 11. The full structure, $[Fe_4S_4(STbt)_4]$ (Tbt = 2,4,6-tris{bis(trimethylsilyl)methyl}phenyl), is available at the Cambridge Crystal-

Table S2: Cartesian coordinates [Å] of (**2**).

Atom	X	Y	Z
Fe1	-0.02439996	-1.37009996	1.00249279
Fe2	0.02440004	1.37010004	1.00249279
Fe3	1.37010004	-0.02439996	-1.00240721
Fe4	-1.37009996	0.02440004	-1.00240721
S5	-1.76939996	0.01250004	1.24519279
S6	0.01250004	1.76940004	-1.24520721
S7	-0.01249996	-1.76939996	-1.24520721
S8	1.76940004	-0.01249996	1.24519279
S9	3.08570004	0.25770004	-2.36080721
S10	0.25770004	-3.08569996	2.36079279
S11	-0.25769996	3.08570004	2.36079279
S12	-3.08569996	-0.25769996	-2.36080721
C13	-1.08718996	-4.07672696	2.72580679
C14	1.08719004	4.07672704	2.72580679
C15	4.07672704	-1.08718996	-2.72582121
C16	-4.07672796	1.08719004	-2.72582121
H17	-0.77927696	-4.87111196	3.40408679
H18	-1.86296796	-3.47618996	3.19850679
H19	-1.47580696	-4.51400696	1.80728479
H20	0.77927804	4.87111204	3.40408679
H21	1.86296804	3.47619004	3.19850579
H22	1.47580704	4.51400704	1.80728379
H23	4.87108804	-0.77928696	-3.40413321
H24	4.51404004	-1.47578196	-1.80730321
H25	3.47618204	-1.86298496	-3.19848321
H26	-4.87108796	0.77928604	-3.40413321
H27	-4.51403996	1.47578204	-1.80730321
H28	-3.47618296	1.86298504	-3.19848321

lographic Data Center (CCDC-1559014). In our model the (Tbt) groups have been replaced by methyl groups (-CH3) for simplicity (at the arbitrary distance of 1.71 Å from the peripheral S atoms). In applying this change no bonds of the core cuboid have been modified and no geometry optimization applied to avoid any geometrical artifact introduced by the method chosen for the geometry optimizations. Instead, the experimentally determined structure for the core cuboid has been retained with the only difference being the positioning of the methyl groups. It has been shown that the electronic structure of tetrahedral transition

metal complexes is very sensitive to the orientation of the second and even the third coordination sphere.[12–14] Ligand field effects crucially impact the splitting of the 3d orbitals at the metal center. For Fe(III) cubanes here presented, modifications of the 3d orbital splittings would certainly affect the high energy portion of the electronic spectrum, where states with local $S \neq 5/2$ exist and ligand field effects play more prominent role. We recommend Reference 15 for a deeper analysis of this aspect. However, local ligand field effects play a smaller role in the coupling of local $S_{\mathrm{local}} = 5/2$ spins across the magnetic sites in the low energy spectrum. Structure (**2**) shows two short and four long bonds.

Table S3: Selected bond lengths [Å] of (**1**) and (**2**).

Atoms		Distance in (**1**)	Distance in (**2**)
Fe1	Fe2	2.85	2.74
Fe3	Fe4	2.85	2.74
Fe1	Fe3	2.75	2.79
Fe2	Fe4	2.75	2.79
Fe1	Fe4	2.75	2.79
Fe2	Fe3	2.75	2.79
Fe1	S5	2.34	2.24
Fe1	S7	2.20	2.28
Fe1	S8	2.33	2.26
Fe2	S5	2.33	2.26
Fe2	S6	2.21	2.28
Fe2	S8	2.32	2.24
Fe3	S6	2.32	2.26
Fe3	S7	2.33	2.24
Fe3	S8	2.21	2.28
Fe4	S5	2.20	2.28
Fe4	S6	2.33	2.24
Fe4	S7	2.34	2.26
Fe1	S10	2.25	2.21
Fe2	S11	2.24	2.21
Fe3	S9	2.25	2.21
Fe4	S12	2.25	2.21

S4 Basis set, orbitals and active spaces

The *complete active space* (CAS) is a simple and natural concept to tackle multi-reference systems in chemistry.[16–20] The *active space* is a list of “critical” orbitals with their electrons, for which a *complete* many-body expansion is generated and its expansion coefficients (CI amplitudes) are variationally optimized. When orbitals are self-consistently (SCF) optimized together with the CI coefficients, under the field generated by the multiconfigurational wave function, the method is referred to as CASSCF. The active space orbitals used in our work were generated by performing a Restricted Open Shell Hartree-Fock (ROHF) calculation for the highest spin state (S=10) of (**1**) and (**2**). This calculation is identical to a CASSCF(20e,20o) optimization of the high $S = 10$ spin state, with the active space consisting of the twenty 3 d valence orbitals of the iron centers and their electrons. Due to the choice of the active space and the targeted spin, this procedure is, in practice, a single-configurational optimization. In our paradigm, the resulting active orbitals undergo an invariant orbital transformation, namely a Pipek-Mezey[21] localization, followed by their sorting in *atom-separated* order. A second invariant MO transformation is performed, only for constructing the (44e,32o) active spaces, that involves the inactive orbitals of the CAS(20e,20o). This second transformation consists of a Pipek-Mezey[21] localization of the inactive MOs, and it is necessary to identify the doubly occupied (inactive) MOs mostly with the character of the 3 p AOs of the bridging sulfur atoms. In our CAS(44e,32o) calculations the localized orbitals of the S atoms follow (in order) the MOs of the iron centers. The resulting orbitals are summarized in Figure 7. Orbitals of the peripheral thiolate groups are crucial when accounting for ligand field effects, for example for accurate estimates of the local $10Dq$ values. In turn, these strongly affect the spin-state energy separations in mixed-valence PNTM clusters. This aspect has been discussed already in the literature.[8,22,23] Of particular relevance is the angular overlap model (AOM) analysis of Reference 23, where the bridging sulfide ligand and peripheral thiolate ligand mixing is discussed. In the present work, however, only the low-energy spectrum of all-ferric metal centers has been considered, for which ligand field

effects are not as critical as for the mixed-valent case, and could be considered of high order and only of quantitative impact. Thus, for the present study peripheral orbitals have not been included in the active space.

Generally contracted atomic natural orbitals (ANO-RCC) basis sets[24,25] have been employed, obtained from the Fe(21s15p10d6f4g2h), S(17s12p5d4f2g), C(14s9p4d3f2g), and H(8s4p3d1f) primitive functions, contracted to Fe(5s4p2d), S(3s2p), C(2s1p) and H(1s), giving a basis set of split-valence double-ζ quality (VDZ) for the iron centers and a minimal basis for all other atoms. Scalar relativistic effects were introduced via second order Douglas-Kroll-Hess integral correction. For all calculations the evaluation of the electron repulsion integrals has been simplified by means of the resolution-of-identity Cholesky decomposition technique,[3] with a decomposition threshold of 10^{-4}a.u.[26] Both (**1**) and (**2**) belong to the D_{2d} point group symmetry. However, all calculations were performed within the C_1 point group symmetry, as the localization procedure utilized here removes any symmetry from the MOs. All calculations were carried within the OpenMolcas chemistry software package.[4]

S5 GUGA-FCIQMC details

Our theoretical arguments have been supported by numerical evidence of the compression and resolution of states for six energetically low-lying singlet states for (**1**) and (**2**) and their highest spin states ($S = 10$). Considering the considerably large size of the active spaces investigated —CAS(20e,20o) and CAS(44e,32o) —all calculations have been performed using the FCIQMC algorithm[3,4,27–35] in its spin-adapted formulation,[36] available in the NECI code.[35] The initiator formulation of FCIQMC has been used,[28,29] with a threshold value of $n_a = 3.0$, together with the semi-stochastic approach,[31,37] with the deterministic subspace consisting of $|\mathcal{D}| = 10000$ most populated CSFs, determined once the calculation reaches variable shift mode. It can be observed in Figure S2 that once the semi-stochastic propagation is started, the fluctuations in energy are sharply reduced. For all calculations, a single

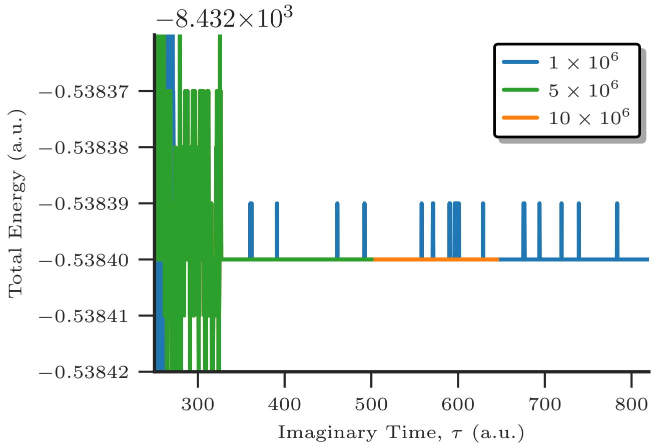

Figure S2: Spin-adapted FCIQMC dynamics for the lowest singlet spin state of compound (**1**) within the CAS(20e,20o) for 1×10^6, 5×10^6, 10×10^6 walkers. A sharp reduction in fluctuations in the energy occurs when the semi-stochastic propagation is started.

CSF has been used as the starting wave function for the FCIQMC dynamics, chosen among the paths of the genealogical branching diagram for each singlet spin state. As discussed in the main text, thanks to the block diagonal structure of the CI Hamiltonian matrix, the choice of the reference CSF determines the state that will be optimized (ground or excited state). The time-step $\Delta\tau$ was found via an automatic search procedure[32] for each simulation, and took typical values in the range $2 - 10 \times 10^{-3}$ a.u. for the CAS(20e,20o) calculations, and $1 - 3 \times 10^{-4}$ a.u. for the CAS(44e,32o).

Within the FCIQMC method, the total number of walkers is a crucial parameter that determines the computational costs as well as the accuracy of the final energies and wave functions. For the CAS(20e,20o) calculations convergence with respect to the number of walkers was tested by using three walker populations, namely 1×10^6, 5×10^6 and 10×10^6 walkers. The corresponding dynamics for the ground state of (**1**) are reported in Figure S2.

The effect of the MO transformation is evident. We have reached μ-Hartree convergence already with 5×10^6 walker population used with negligible stochastic fluctuations.

For the CAS(44e,32o) the walker population plays a more important role as shown in Figure S3. The lowest-to-highest spin energy gap as function of the walker population is also

summarized in Table S4.

Table S4: Lowest-to-highest spin energy gap of (**1**), for the (44e,32o) active space and a varying number of walkers in the FCIQMC dynamics.

Walker Population [$\times 10^6$]	Spin Gap [meV]
1	185
10	164
20	158
50	153
100	151

The energy gap between the lowest and the highest singlet spin state reduces as the walker population is increased, from 185 meV with 1×10^6 walkers to 151 meV when using 100×10^6 walkers. Although the qualitative behavior is unchanged at lower walker population, allowing us to draw the similar conclusions, quantitatively the walker population plays a role. This effect is to be related to the charge-transfer excitations. Our wave function is highly compressed with respect to the leading configurations, namely the spin-flip configurations. However, the charge-transfer excitations need to be accounted for and FCIQMC does so by distributing walkers in the available FCI space. The space sampled increases and its

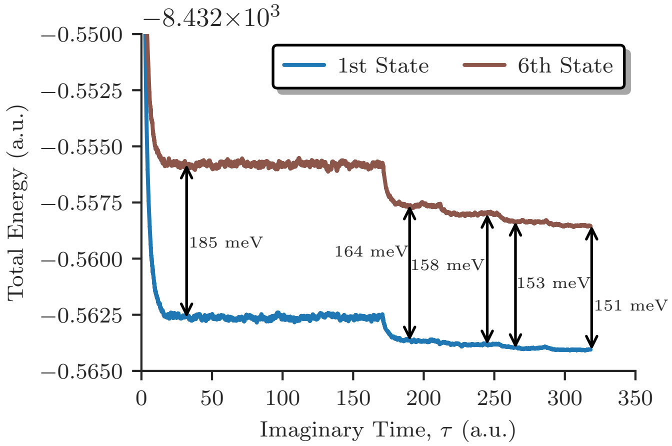

Figure S3: Spin-adapted FCIQMC dynamics for the lowest and highest singlet spin states of compound (**1**) within the CAS(44e,32o) active space for 1×10^6, 10×10^6, 20×10^6, 50×10^6 and 100×10^6 walkers. The colors used for the trajectories correspond to the ones utilized in Figure 3 to identify the leading components of the six singlet states.

Table S5: Leading CSFs for the lowest and highest singlet states of (**1**) and (**2**) within the CAS(20e,20o). Symbols u and d refers to an up (u) or down (d) coupling of the spin in a cumulative manner. The weight is given as percent square of the coefficients of the normalized FCI wave function.

Fe_A	Fe_B	Fe_C	Fe_D	Weight [%]
		(**1**) $S_{int} = 5$ (Ground State)		
uuuuu	uuuuu	ddddd	ddddd	96.0
uuuu0	uuuuu	ddddd	ddd2d	0.1
uuuu2	uuuuu	ddddd	ddd0d	0.1
uuuu2	uuuuu	ddd0d	ddddd	0.1
uuuu0	uuuuu	ddd2d	ddddd	0.1
		(**1**) $S_{int} = 0$		
uuuuu	ddddd	uuuuu	ddddd	96.2
uuu2u	ddd0d	uuuuu	ddddd	0.1
uuu0u	ddd2d	uuuuu	ddddd	0.1
uuuuu	ddddd	uuuu2	dddd0	0.1
uuuuu	ddddd	uuuu0	dddd2	0.1
		(**2**) $S_{int} = 0$ (Ground State)		
uuuuu	ddddd	uuuuu	ddddd	96.1
uuuu0	dd2dd	uuuuu	ddddd	0.2
uuuu2	dd0dd	uuuuu	ddddd	0.2
uuuuu	ddddd	uu0uu	dddd2	0.2
uuuuu	ddddd	uu2uu	dddd0	0.2
		(**2**) $S_{int} = 5$		
uuuuu	uuuuu	ddddd	ddddd	96.6
uuuu2	uuuuu	dddd0	ddddd	0.1
uuuuu	uu2uu	ddddd	ddd0d	0.1
uu0uu	uuuuu	ddddd	dddd2	0.1
uuuuu	uuu0u	dd2dd	ddddd	0.1

representation gets more accurate as the population is enlarged, leading to improved accuracy as we grow the walker population.

In Table S5 we offer a list of the leading CSFs for the lowest and highest singlet states investigated for (**1**) and (**2**). As discussed in the main text, in the atom-separated ordering, the lowest and highest singlet state wave functions are largely single reference. The large weight of $\sim 96\%$ on the CSF chosen as reference proofs this property. All other CSFs with weights

$\sim 0.1\%$ represent metal-to-metal charge-transfer configurations. In the larger CAS(44e,32o) a very similar wave function landscape exists, except that ligand-to-metal charge-transfer excitations appear, with weight comparable to the metal-to-metal CT. Due to the presence in the FCI wave function of the ligand-to-metal CT excitations, the weights at the reference CSF in the CAS(44e,32o) are reduced compared to the weight of the CAS(20e,20o). For all states the weight is $\sim 87\%$.

S6 Local spin measurements

To analyze the spin structure in our calculations we measured the local spin defined as[8,38]

$$\hat{S}_A^\alpha = \sum_{i\in A} s_i^\alpha, \quad \hat{\mathbf{S}}_A \cdot \hat{\mathbf{S}}_A = \sum_\alpha \hat{S}_A^\alpha \hat{S}_A^\alpha, \quad \text{with} \quad \alpha \in \{x, y, z\} \tag{1}$$

where s_i^α are the spin operators of localized orbital i in the domain A. A can include all the localized orbitals of an iron center, but also localized orbitals of multiple iron atoms for cumulative spin measurements.

CSFs in the GUGA approach are sequentially coupled to remain spin eigenfunctions with each added spatial orbital. Consequently each CSF $|D_k\rangle$ is an spin-eigenfunction up to every spatial orbital $j \leq n$ with a specific spin-quantum number $S_j^{(k)}$, $\hat{\mathbf{S}}_j^2 |D_i\rangle = S_j^{(k)}(S_j^{(k)}+1)|D_i\rangle$ and n being the total number of spatial orbitals. Hence, measuring the local spin (Equation 1) is straightforward in GUGA-FCIQMC as long as the targeted orbitals $i \in A$ are ordered subsequently from the beginning as

$$\langle\Psi|\hat{\mathbf{S}}_A^2|\Psi\rangle = \sum_k c_k^* c_k \langle D_k|\hat{\mathbf{S}}_A^2|D_k\rangle = \sum_k c_k^* c_k S_A^{(k)} \left(S_A^{(k)} + 1\right), \tag{2}$$

with $|\Psi\rangle = \sum_k c_k |D_k\rangle$ being the stochastically sampled wave function in FCIQMC.

References

(1) Davidson, E. R. the Iterative Calculation of a Few of the Lowest Eigenvalues and Corresponding Eigenvectors of Large Real-Symmetric Matrices. *J. Comput. Phys.* **1975**, *17*, 87–94.

(2) Lanczos, C. an Iteration Method for the Solution of the Eigenvalue Problem of Linear Differential and Integral Operators. *J. Nat. Bur. Stand.* **1950**, *45*, 225–282.

(3) Aquilante, F. et al. Molcas 8: New Capabilities for Multiconfigurational Quantum Chemical Calculations Across the Periodic Table. *J. Comput. Chem.* **2016**, *37*, 506–541.

(4) Fdez. Galván, I. et al. OpenMolcas: From Source Code to Insight. *J. Chem. Theory Comput.* **2019**, *15*, 5925–5964.

(5) Li Manni, G.; Dobrautz, W.; Alavi, A. Compression of Spin-Adapted Multiconfigurational Wave Functions in Exchange-Coupled Polynuclear Spin Systems. *Journal of Chemical Theory and Computation* **2020**, *16*, 2202–2215.

(6) Olsen, J.; Roos, B. O.; Jørgensen, P.; Jensen, H. J. A. Determinant Based Configuration Interaction Algorithms for Complete and Restricted Configuration Interaction Spaces. *J. Chem. Phys.* **1988**, *89*, 2185–2192.

(7) Ma, D.; Li Manni, G.; Gagliardi, L. The generalized active space concept in multiconfigurational self-consistent field methods. *J. Chem. Phys.* **2011**, *135*, 044128.

(8) Sharma, S.; Sivalingam, K.; Neese, F.; Chan, G. K.-L. Low-energy spectrum of iron-sulfur clusters directly from many-particle quantum mechanics. *Nat. Chem.* **2014**, *6*, 927–933.

(9) Weigend, F.; Ahlrichs, R. Balanced Basis Sets of Split Valence, Triple Zeta Valence

and Quadruple Zeta Valence Quality for H to Rn: Design and Assessment of Accuracy. *Phys. Chem. Chem. Phys.* **2005**, *7*, 3297–3305.

(10) Averill, B. A.; Herskovitz, T.; Holm, R. H.; Ibers, J. A. Synthetic analogs of the active sites of iron-sulfur proteins. II. Synthesis and structure of the tetra[mercapto-.mu.3-sulfido-iron] clusters, [Fe4S4(SR)4]2-. *Journal of the American Chemical Society* **1973**, *95*, 3523–3534.

(11) Moula, G.; Matsumoto, T.; Miehlich, M. E.; Meyer, K.; Tatsumi, K. Synthesis of an All-Ferric Cuboidal Iron-Sulfur Cluster [$Fe^{III}{}_4S_4(SAr)_4$]. *Angewandte Chemie International Edition* **2018**, *57*, 11594–11597.

(12) Suturina, E. A.; Maganas, D.; Bill, E.; Atanasov, M.; Neese, F. Magneto-Structural Correlations in a Series of Pseudotetrahedral [CoII(XR)4]2âĂŞ Single Molecule Magnets: An ab Initio Ligand Field Study. *Inorganic Chemistry* **2015**, *54*, 9948–9961.

(13) Suturina, E. A.; Nehrkorn, J.; Zadrozny, J. M.; Liu, J.; Atanasov, M.; Weyhermüller, T.; Maganas, D.; Hill, S.; Schnegg, A.; Bill, E.; Long, J. R.; Neese, F. Magneto-Structural Correlations in Pseudotetrahedral Forms of the [Co(SPh)4]2âĂŞ Complex Probed by Magnetometry, MCD Spectroscopy, Advanced EPR Techniques, and ab Initio Electronic Structure Calculations. *Inorganic Chemistry* **2017**, *56*, 3102–3118.

(14) Spiller, N.; Chilkuri, V. G.; DeBeer, S.; Neese, F. Sulfur vs. Selenium as Bridging Ligand in Di-Iron Complexes: A Theoretical Analysis. *European Journal of Inorganic Chemistry* **2020**, *2020*, 1525–1538.

(15) Chilkuri, V. G.; DeBeer, S.; Neese, F. Ligand Field Theory and Angular Overlap Model Based Analysis of the Electronic Structure of Homovalent Iron–Sulfur Dimers. *Inorganic Chemistry* **2019**, *59*, 984–995.

(16) Roos, B. O.; Taylor, P. R.; Siegbahn, P. E. M. A Complete Active Space SCF Method

(CASSCF) Using a Density Matrix Formulated Super-CI Approach. *Chem. Phys.* **1980**, *48*, 157–173.

(17) Roos, B. O. The Complete Active Space SCF Method in a Fock-Matrix-Based Super-CI Formulation. *Int. J. Quantum Chem.* **1980**, *18*, 175–189.

(18) Siegbahn, P. E. M.; Heiberg, A.; Roos, B. O.; Levy, B. A Comparison of the Super-CI and the Newton-Raphson Scheme in the Complete Active Space SCF Method. *Phys. Scr.* **1980**, *21*, 323–327.

(19) Siegbahn, P. E. M.; Almlöf, J.; Heiberg, A.; Roos, B. O. The Complete Active Space SCF (CASSCF) Method in a Newton–Raphson Formulation with Application to the HNO Molecule. *J. Chem. Phys.* **1981**, *74*, 2384–2396.

(20) Roos, B. O. *Advances in Chemical Physics: Ab Initio Methods in Quantum Chemistry Part 2*; 2007; Vol. 69; pp 399–445.

(21) Pipek, J.; Mezey, P. G. A Fast Intrinsic Localization Procedure Applicable for Ab Initio and Semiempirical Linear Combination of Atomic Orbital Wave Functions. *J. Chem. Phys.* **1989**, *90*, 4916–4926.

(22) Cho, D.; Rouxel, J. R.; Mukamel, S.; Chan, G. K.-L.; Li, Z. Stimulated X-ray Raman and Absorption Spectroscopy of Iron–Sulfur Dimers. *The Journal of Physical Chemistry Letters* **2019**, *10*, 6664–6671.

(23) Chilkuri, V. G.; DeBeer, S.; Neese, F. Ligand Field Theory and Angular Overlap Model Based Analysis of the Electronic Structure of Homovalent IronâĂŞSulfur Dimers. *Inorganic Chemistry* **2020**, *59*, 984–995.

(24) Widmark, P.-O.; Malmqvist, P.-Å.; Roos, B. O. Density Matrix Averaged Atomic Natural Orbital (ANO) Basis Sets for Correlated Molecular Wave Functions. *Theor. Chem. Acc.* **1990**, *77*, 291–306.

(25) Roos, B. O.; Lindh, R.; Malmqvist, P.-Å.; Veryazov, V.; Widmark, P.-O. Main Group Atoms and Dimers Studied with a New Relativistic ANO Basis Set. *J. Phys. Chem. A* **2004**, *108*, 2851–2858.

(26) Aquilante, F.; Pedersen, T. B.; Lindh, R.; Roos, B. O.; de Merás, A. S.; Koch, H. Accurate Ab Initio Density Fitting for Multiconfigurational Self-Consistent Field Methods. *J. Chem. Phys.* **2008**, *129*, 024113.

(27) Booth, G. H.; Thom, A. J. W.; Alavi, A. Fermion Monte Carlo without fixed nodes: A game of life, death, and annihilation in Slater determinant space. *J. Chem. Phys.* **2009**, *131*, 054106.

(28) Cleland, D. M.; Booth, G. H.; Alavi, A. Survival of the Fittest: Accelerating Convergence in Full Configuration-Interaction Quantum Monte Carlo. *J. Chem. Phys.* **2010**, *132*, 041103.

(29) Cleland, D.; Booth, G. H.; Alavi, A. A Study of Electron Affinites Using the Initiator Approach to Full Configuration Interaction Quantum Monte Carlo. *J. Chem. Phys.* **2011**, *134*, 024112.

(30) Overy, C.; Booth, G. H.; Blunt, N. S.; Shepherd, J. J.; Cleland, D.; Alavi, A. Unbiased Reduced Density Matrices and Electronic Properties from Full Configuration Interaction Quantum Monte Carlo. *J. Chem. Phys.* **2014**, *141*, 244117.

(31) Blunt, N. S.; Smart, S. D.; Kersten, J. A.-F.; Spencer, J. S.; Booth, G. H.; Alavi, A. Semi-Stochastic Full Configuration Interaction Quantum Monte Carlo: Developments and Application. *J. Chem. Phys.* **2015**, *142*, 184107.

(32) Booth, G. H.; Smart, S. D.; Alavi, A. Linear-Scaling and Parallelisable Algorithms for Stochastic Quantum Chemistry. *Mol. Phys.* **2014**, *112*, 1855–1869.

(33) Blunt, N. S.; Alavi, A.; Booth, G. H. Krylov-Projected Quantum Monte Carlo Method. *Phys. Rev. Lett.* **2015**, *115*, 050603.

(34) Li Manni, G.; Smart, S. D.; Alavi, A. Combining the Complete Active Space Self-Consistent Field Method and the Full Configuration Interaction Quantum Monte Carlo within a Super-CI Framework, with Application to Challenging Metal-Porphyrins. *J. Chem. Theory Comput.* **2016**, *12*, 1245–1258.

(35) Guther, K. et al. NECI: N-Electron Configuration Interaction with an emphasis on state-of-the-art stochastic methods. *The Journal of Chemical Physics* **2020**, *153*, 034107.

(36) Dobrautz, W.; Smart, S. D.; Alavi, A. Efficient formulation of full configuration interaction quantum Monte Carlo in a spin eigenbasis via the graphical unitary group approach. *J. Chem. Phys.* **2019**, *151*, 094104.

(37) Petruzielo, F. R.; Holmes, A. A.; Changlani, H. J.; Nightingale, M. P.; Umrigar, C. J. Semistochastic Projector Monte Carlo Method. *Phys. Rev. Lett.* **2012**, *109*, 230201.

(38) Ramos-Cordoba, E.; Matito, E. Local Descriptors of Dynamic and Nondynamic Correlation. *Journal of Chemical Theory and Computation* **2017**, *13*, 2705–2711.
